# Umbilical Cord Blood Gasometry and pH as Key Regulators of Growth Factor Expression Profile in Umbilical Cord-Derived Mesenchymal Stromal Cells (UC-MSCs)

**DOI:** 10.3390/cells15121076

**Published:** 2026-06-13

**Authors:** Dominika Przywara, Wiktor Babiuch, Alicja Petniak, Małgorzata Wasilewska, Jarosław Krzyżanowski, Monika Czuba, Arkadiusz Krzyżanowski, Adrianna Kondracka, Janusz Kocki, Paulina Gil-Kulik

**Affiliations:** 1 Department of Clinical Genetics, Medical University of Lublin, 11 Radziwillowska Str., 20-080 Lublin, Poland; dprzywara17@gmail.com (D.P.); alicja.petniak@umlub.pl (A.P.); monika.czuba@umlub.pl (M.C.); janusz.kocki@umlub.pl (J.K.); 2 Doctoral School, Medical University of Lublin, 20-093 Lublin, Poland; 3 Student Scientific Society of Clinical Genetics, Medical University of Lublin, 20-080 Lublin, Poland; babiuchwik@gmail.com; 4 Department of Physical Chemistry, Institute of Chemical Sciences, Faculty of Chemistry, Maria Curie-Sklodowska University, Maria Curie-Sklodowska Sq. 3, 20-031 Lublin, Poland; malgorzata.wasilewska@mail.umcs.pl; 5 Department of Gynecology and Obstetrics, The Independent Public Hospital No. 4, 20-090 Lublin, Poland; krzyzanowski.jaroslaw@tlen.pl; 6 Department of Obstetrics and Pathology of Pregnancy, Medical University of Lublin, 11 Staszica Str., 20-081 Lublin, Poland; arkadiusz.krzyzanowski@umlub.pl (A.K.); adrianna.kondracka@umlub.pl (A.K.)

**Keywords:** umbilical cord mesenchymal stromal cells (UC-MSCs), umbilical cord blood pH, transcriptomics, neonatal asphyxia, trophic factors, perinatal microenvironment, cell proliferation, regenerative medicine

## Abstract

**Highlights:**

**What are the main findings?**

- Umbilical cord blood pH acts as a molecular switch for UC-MSCs, where acidic conditions (pH < 7.35) trigger a transcriptomic shift from a proliferative to a structural–migratory phenotype characterized by intensive extracellular matrix remodeling.
- Perinatal metabolic stress, hypercapnia, and threatened neonatal asphyxia significantly reduce the expression of key trophic and neuroprotective factors, specifically the *EGF*, *FGFR1*, *NGF*, and *IGF1* axes.

**What are the implications of the main findings?**

- The acid–base status and gas parameters of umbilical cord blood serve as objective indicators of the “biological quality” and therapeutic potential of isolated stromal cells.
- Clinical screening of the neonatal microenvironment is essential for the standardization and selection of UC-MSCs to ensure optimal paracrine and neurotrophic efficacy in regenerative medicine applications.

**Abstract:**

Umbilical cord mesenchymal stromal cells (UC-MSCs) are a key element of regenerative medicine due to their ability to secrete growth factors that stimulate proliferation and angiogenesis, and modulate the inflammatory response. Despite their widespread use, the influence of the perinatal microenvironment on their biological properties remains poorly understood. The aim of this study was to assess the influence of pH and blood gas parameters in umbilical cord blood on the global transcriptomic profile of UC-MSCs and to analyze the correlation between the metabolic status of the newborn and the expression of key trophic factors: *EGF*, *FGF2*, *FGFR1*, *FGFR3*, *GDNF*, *HGF*, *IGF1*, *NES*, *NGF*, and *PGF.* Methods: The study was conducted in two stages. In the first phase, transcriptomic screening was performed using Affymetrix HuGene 2.0 ST microarray on cells isolated from three environmental groups defined by cord blood pH: acidic (pH < 7.35), physiological (7.35–7.39), and alkaline (pH ≥ 7.4). In the second phase, the results were validated using qPCR on an expanded study group (N = 50). Gene expression levels (RQ) were related to blood gas parameters (pH, pCO_2_, pO_2_, cHCO_3_) and the presence of clinical features of threatened neonatal asphyxia. Results: Microarray analysis revealed that environmental pH acts as a molecular phenotypic switch. Under low pH conditions (<7.35), a shift in cell profile from proliferative to structural–migratory was observed. Significant overexpression of genes responsible for extracellular matrix (ECM) organization and adhesion (e.g., *COMP*, *DCN*, *LUM*, *FMOD*) was observed, while pathways related to cell cycle and cell division (↓*CDK1*, *AURKA*, *TOP2A*) were downregulated. qPCR validation confirmed these observations, demonstrating a strong positive correlation between blood pH and the expression of regenerative mediators: *FGFR1* (r = 0.28), *EGF* (r = 0.30), *NGF* (r = 0.39), and *IGF1* (r = 0.30). A negative correlation was also found between carbon dioxide pressure (pCO_2_) and the expression of *NGF*, *FGFR1*, and *EGF.* A significant clinical finding was that in newborns diagnosed with threatened asphyxia, *EGF*, *FGFR1*, and *NGF* gene expression was significantly reduced, indicating impaired trophic potential of the cells in response to metabolic stress. Conclusions: These results indicate that cord blood gas parameters are critical regulators of the genetic activity of UC-MSCs. Metabolic and respiratory acidosis not only inhibit the cells’ proliferative potential but also force them into a matrix remodeling mode, permanently modifying their transcriptomic profile. This suggests that the neonatal acid–base status may serve as an objective indicator of the “biological quality” of isolated stromal cells, which has significant implications for their future applications in cell therapies.

## 1. Introduction

Mesenchymal stromal cells (MSCs) are multipotent cells that, primarily through their paracrine activity, are capable of performing diverse therapeutic functions. MSCs secrete a broad spectrum of growth factors, cytokines, and proteins that confer pro-angiogenic, anti-apoptotic, and anti-inflammatory properties, while also stimulating cell growth and proliferation. Consequently, MSCs possess a significant capacity to support complex regenerative processes [1,2].

While MSCs are widely present in the adult organism, fetal tissues—such as the placenta, umbilical cord blood, and Wharton’s jelly—represent a superior source. Fetal MSCs are characterized by higher proliferative capacity and greater plasticity compared with MSCs derived from mature tissues [1,3]. Furthermore, as the number and quality of MSCs in tissues decrease with age, fetal-derived cells have become a primary focus for applications in regenerative medicine, including wound healing, musculoskeletal disorders, and neurological diseases [4,5,6].

The functional plasticity of MSCs is strictly regulated by their microenvironmental niche, where physicochemical factors such as oxygen tension and pH play a pivotal role. In the context of the umbilical cord, these factors are highly dynamic and reflect the metabolic status of the fetus during the transition from intrauterine to extrauterine life. MSCs constitute a highly heterogeneous population, and their properties are influenced by donor-specific factors, including age, chronic diseases, and hematological parameters [1,2,7,8].

Umbilical cord blood gasometry, particularly pH and pCO_2_, is considered the gold standard for assessing fetal metabolic status and the degree of intrapartum stress. Acid–base disturbances, often resulting from transient hypoxia during labor, may act as potent biological stimuli that trigger rapid adaptive responses in the surrounding stromal cell populations, potentially altering their therapeutic efficacy [2,7,8].

Previous studies have demonstrated that maternal conditions, such as diabetes and obesity, can impair the proliferation and anti-inflammatory properties of MSCs [7,9,10]. Additionally, our prior research suggested that umbilical cord blood hematological and biochemical parameters significantly influence the anti-inflammatory and pluripotent characteristics of Wharton’s jelly-derived MSCs (UC-MSCs) [2,11,12,13]. Despite these findings, the global transcriptomic response of UC-MSCs to the acid–base status of the umbilical cord niche remains poorly characterized.

In this study, we employed a dual approach, combining global transcriptomic profiling via microarray with targeted qPCR validation. We selected a specific panel of genes—*EGF*, *FGF2*, *GDNF*, *HGF*, *IGF1*, *NES*, *NGF*, and *PGF*—along with receptors *FGFR1* and *FGFR3*, as they represent the fundamental functional axes of MSC-mediated regeneration [14,15,16,17,18,19,20,21,22,23,24,25,26,27,28,29,30,31,32,33]. Specifically, *EGF*, *FGF2*, *IGF1*, and *HGF* were selected for their critical roles in cell proliferation and mitogenesis, while *NGF*, *GDNF*, and *NES* serve as key markers of neuroprotection and nervous system regeneration. Furthermore, *PGF* and *FGF2* were included due to their essential involvement in pro-angiogenic processes [18,19,20,21,22,23,24,25,26,27,28,29,30,31,32,33].

The aim of this study was to evaluate the expression of these selected genes in UC-MSCs and to analyze how their levels correlate with maternal health status, the occurrence of threatened neonatal asphyxia, and umbilical cord blood gasometry parameters. We hypothesize that UC-MSCs undergo functional reprogramming in response to the perinatal microenvironment, shifting between proliferative and adaptive phenotypes. This study seeks to define the molecular signature of this response, providing new insights into the selection of optimal donor material for regenerative medicine.

Consequently, our findings suggest that baseline acid–base status and cord blood gas parameters may serve as potential indirect indicators of transcriptional activity and early screening parameters for the biological quality of isolated UC-MSCs. However, it must be emphasized that these observations are limited to in vitro data at the transcript level, and further validation using functional and protein assays is necessary to establish a definitive and biological classification.

## 2. Materials and Methods

### 2.1. Materials

The material used for analysis consisted of UC-MSCs. Umbilical cords were collected by a physician immediately after delivery from 50 women who gave birth at the Clinic of Obstetrics and Pregnancy Pathology of the Independent Public Clinical Hospital No. 1 in Lublin. Prior to delivery, each woman was informed about the aim of the study and provided written informed consent to participate. The study was conducted with the approval of the Bioethics Committee of the Medical University of Lublin pursuant to resolution no. KE-0254/7/01/2023. The characteristics of the study group are presented in Table 1 and Table 2.

To clearly determine the overlap between perinatal acid–base status and clinical diagnosis, the distribution of neonatal threatened asphyxia cases within defined pH groups was analyzed. In the extended validation cohort (N = 50), all 5 cases (100%) of clinically confirmed threatened asphyxia were strictly from the acidic microenvironment subgroup (pH < 7.35). More specifically, the exact cord blood pH values recorded for these 5 neonates with asphyxia were 7.28–7.31, indicating a significant clustering of clinical asphyxia cases within the narrow range of metabolic and respiratory acidosis, whereas no asphyxia cases occurred in the physiological (pH = 7.35–7.39) and alkaline (pH ≥ 7.40) subgroups.

To provide a detailed understanding of the baseline characteristics of the cohort presented in Table 1, the entire population (N = 50) was divided into physiological cord blood pH groups. Specifically, 25 samples (50%) were classified into the acidic microenvironment group (pH < 7.35), 18 samples (36%) fell into the physiological reference range (pH = 7.35–7.39), and the remaining 7 samples (14%) fell into the alkaline microenvironment group (pH ≥ 7.40).

To clarify the relationship between study stages, the 6 primary cord blood samples used for the global microarray transcriptome screen constituted an integral subset of the entire validation cohort (N = 50) examined in this study. Regarding their baseline acid–base stratification, these 6 discovery samples were evenly distributed across experimental categories (*n* = 2 per pH group), consisting of 2 samples from the acidic microenvironment group (pH < 7.35), 2 samples from the physiological reference group (pH = 7.35–7.39), and 2 samples from the alkaline microenvironment group (pH ≥ 7.40).

### 2.2. Methods

#### 2.2.1. Assessment of Threatened Neonatal Asphyxia

At-risk neonatal hypoxia (at-risk fetal hypoxia) was clinically assessed and diagnosed by the attending physicians based on a multiparameter protocol including intrapartum cardiotocography (CTG) and paired umbilical cord blood gas analysis after delivery. According to the current guidelines of the Polish Society of Gynecologists and Obstetricians [34] and standard consensus frameworks (FIGO, NICE, ACOG), the decisive intrapartum CTG criteria indicating a high risk of decompensated metabolic acidosis were: repetitive late decelerations occurring in 50% of contractions; prolonged variable decelerations lasting >60 s; baseline bradycardia (<110 beats per minute) for >10 min, indicating fetal compensatory failure; limited baseline variability (silent oscillation) characterized by an amplitude of variability <5 beats per minute for >40 min. Clinical findings, such as the presence of meconium-stained amniotic fluid, have been noted as risk factors. Furthermore, computerized CTG analysis (Dawes–Redman system) was used to assess short-term variability (STV) for prenatal risk stratification before the onset of active labor. STV values below 3–4 ms have been defined by clinicians as concerning, representing chronic fetal hypoxia, autonomic dysregulation, and increased metabolic risk, particularly in premature or growth-restricted fetuses [34,35,36,37].

#### 2.2.2. Evaluation of Blood Gasometric and Hematological Parameters of Umbilical Cord Blood

Cord blood gases and hematological parameters were determined using standard, validated clinical procedures at the point of care. To ensure optimal sample quality, tissue integrity, and neonatal safety, cord blood collection was performed immediately after delivery and double cord clamping, before placental expulsion [38,39]. Under sterile conditions, the isolated cord segment was thoroughly cleaned with an antiseptic solution. To ensure maximum metabolic accuracy and prevent cross-contamination between compartments, paired blood samples were systematically collected in a sequential manner. Blood from the umbilical artery (UA), which precisely reflects the metabolic and acid–base status of fetal tissues, was prioritized and collected first by inserting a needle into one of the smaller, thick-walled umbilical arteries at a tight 30-degree angle. Umbilical venous blood (UV), reflecting maternal-placental homeostasis, was then collected from the larger, thin-walled umbilical vein. For each clinical reading, a volume of 1–2 mL of whole blood was collected into pre-heparinized syringes. Any air bubbles were immediately removed, and the syringes were hermetically sealed, clearly labeled (UA or UV), and immediately sent to the laboratory. All blood gas analyses were performed within 15–30 min after delivery using an automated ABL90 FLEX analyzer (Radiometer, Copenhagen, Denmark) to measure pH, carbon dioxide partial pressure (pCO_2_), oxygen partial pressure (pO_2_), and true bicarbonate concentration (cHCO_3_). Concurrent hematology profiling—including white blood cell (WBC) count, red blood cell (RBC) count, hemoglobin concentration (HGB), hematocrit (HCT), mean corpuscular volume (MCV), mean corpuscular hemoglobin (MCH), mean corpuscular hemoglobin concentration (MCHC), and platelet count (PLT)—was performed using fully validated, automated hematology platforms in strict compliance with current quality control guidelines for point-of-care laboratory medicine.

#### 2.2.3. Isolation of Mesenchymal Stromal Cells from the Umbilical Cord

After collection, the umbilical cord fragment was transported to the Department of Clinical Genetics of the Medical University of Lublin, where the UC-MSCs isolation procedure was initiated. First, the umbilical cord was rinsed with PBS solution. It was then fragmented and subjected to enzymatic digestion using a type I collagenase (Gibco by Life Technologies, Grand Island, NY, USA) solution (10 mg collagenase, 25 mL PBS, 0.5 mL antibiotic). Digestion was carried out at 37 °C for 3 h with continuous mixing. The isolation of UC-MSCs was performed according to a previously established protocol [2,11,40].

#### 2.2.4. Cell Culture Establishment and Immunophenotype Assessment by Flow Cytometry

Primary cultures of human umbilical cord-derived mesenchymal stromal cells (UC-MSCs) were established using a mechanical tissue dissociation protocol combined with sequential filtration, utilizing reagent kits and laboratory equipment as previously described. Briefly, after a 3-h initial incubation, the obtained umbilical cord tissue sample was diluted with phosphate-buffered saline (PBS) and passed through a 100-μm nylon strainer to thoroughly remove dense extracellular debris. The filtered cell solution was centrifuged, and the resulting cell pellet was resuspended for primary cell culture establishment.

**Figure 1:**
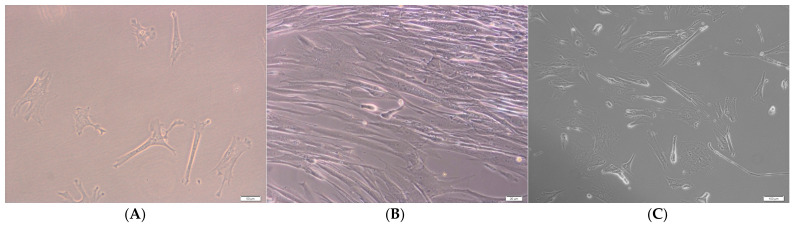
Morphological characterization and growth patterns of primary human MSCs at passage 0 (P0) derived from three independent representative donors (**A**–**C**). Representative inverted light microscopy images illustrate the robust adherence and classical elongated, spindle-shaped fibroblast-like architecture consistently observed across the cohort. (**A**) MSCs at lower confluency showing distinct cell bodies and minimal granularity; (**B**) a higher-magnification view of MSCs at high confluency demonstrating a characteristic parallel, wave-like alignment and dense packing of viable mononuclear cells; (**C**) a wider field-of-view micrograph highlighting uniform cell distribution and lineage homogeneity throughout the culture. Scale bars are embedded directly within the respective micrographs (50 µm for (**A**), 20 µm for (**B**), and 100 µm for (**C**)).

Primary cultures (Figure 1) were grown in Dulbecco’s Modified Eagle Medium (DMEM F-12 Corning, Manassas, VA, USA) supplemented with L-glutamine, high glucose (4.5 g/L), 10% fetal bovine serum (FBS, ATCC, Teddington, UK), and 1% penicillin–streptomycin (Sigma-Aldrich, Jerusalem, Israel) [2,11,12,41]. Importantly, to prevent in vitro cell manipulation artifacts, potential genetic instability, and early replicative senescence associated with prolonged ex vivo expansion, all subsequent molecular and transcriptomic analyses in this study were strictly performed using unpassaged, freshly isolated primary cells at passage 0 (P0). Freshly isolated, unexpanded MSCs have been documented to retain significantly improved homing efficiency and unchanged surface receptor architecture (such as CXCR4 expression) compared to their long-term cultured counterparts [42].

Cultures were incubated at 37 °C with 95% humidity in a controlled atmosphere containing 5% CO_2_ and an adjusted physiological oxygen concentration of 15% O_2_. Although standard laboratory protocols typically use atmospheric air (21% O_2_), this creates a hyperoxic environment compared to the natural physiological stromal cell niche, where tissue physioxia naturally ranges from 5 to 40 mmHg (approximately 1.5–5% O_2_). Culturing UC-MSCs under slightly reduced oxygen tension (15% O_2_ in this model, which represents a short-term, transient physiological limitation) preserves the stromal cell core, protects cells from cumulative oxidative stress, increases proliferation rate, and prevents premature aging without altering standard cell morphology or immunophenotypic lineage characteristics [43,44,45,46,47].

**Figure 2:**
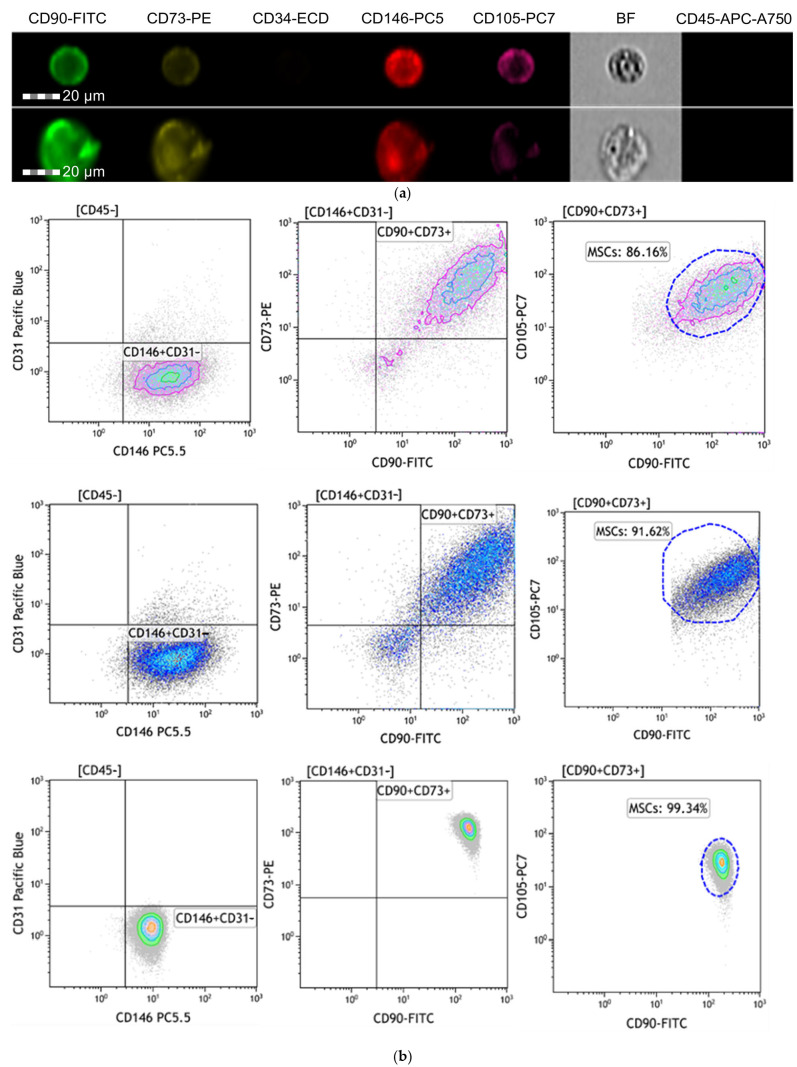
(**a**) Images of individual MSCs, showing the microscopic image (BF) and fluorescence in individual channels, demonstrating the expression of the tested antigens. Analysis and images were taken using a FlowSight f. Amnis flow cytometer. (**b**) Gating strategy and representative multi-donor profiles for quantitative immunophenotypic validation of primary uMSCs at passage 0 (P0) using the Navios flow cytometer (Beckman Coulter) with the DuraClone MSC marker panel (Beckman Coulter, Brea, CA, USA. Flow cytometry profiles from three distinct, independent donors (top, middle, and bottom rows) are shown to illustrate the high technical reproducibility of the isolation protocol. The sequential evaluation cascade demonstrates stringent leukocyte exclusion (CD45-), targeted tracking of the perivascular mesenchymal axis (CD146+/CD31- negative phenotype), and strong commitment to the final multipotent mesenchymal stromal cell lineage (CD90+CD105+CD73+). The final MSC purity across representative batches ranges consistently from 86.16% up to 99.34% of the gated parental populations, confirming high lineage homogeneity prior to downstream transcriptomic analyses. Representative flow cytometry graphs showing the immunophenotype of cells. Quadrant gates (solid lines) were used to determine marker expression. Event density is indicated by color, with warmer colors indicating higher density. Dashed lines indicate the gated MSC population and the corresponding percentage of positive cells.

To maintain high technical consistency and ensure strict quality control throughout the study, immunophenotypic lineage validation was routinely performed on representative, randomly selected batches of isolated primary cultures, according to our highly standardized institutional protocol [2,11,12,41]. Our laboratory has been continuously isolating, culturing, and rigorously evaluating human MSCs since 2011. Because this specific enzymatic framework is deeply established and comprehensively validated in our facility, flow cytometry serves as a periodic methodological Quality Control (QC) gate rather than an analytical endpoint for all 50 individual clinical samples. For the remaining samples, identity was strictly verified via standard plastic adherence and morphological criteria before immediate lysis at Passage 0 (P0) for high-quality RNA isolation. Phenotypic characterization was performed by multispectral flow cytometry using the Amnis FlowSight platform (Amnis Corporation, Seattle, WA, USA) and a Navios cytometer (Beckman Coulter, Bangalore, Karnataka, India) with the DuraClone MSC marker panel (Beckman Coulter, Brea, CA, USA). Single-cell events were strictly gated based on forward scatter intensity (FS INT) versus forward scatter time-of-flight (FS TOF) to exclude doublets and cellular aggregates. Hematopoietic contamination was systematically assessed by filtering for common leukocyte markers, confirming a strong CD45 negativity in the singlet gate. In this nonhematopoietic population, expression of the perivascular mesenchymal marker CD146 and the absence of endothelial lineage markers (CD31-) were verified. As illustrated by three representative, independent donors in Figure 2b, the final triple-positive multipotent mesenchymal stromal cell phenotype (CD90+ CD105+ CD73+) consistently demonstrated high lineage purity, ranging between 86.16% and 99.34% of the gated parental populations. Only primary culture batches meeting these stringent, highly reproducible minimum consensus criteria for MSCs identification were accepted for further transcriptomic and microarray screening.

Based on the historical data and established quality control records of our laboratory, this optimized 3-h collagenase digestion protocol consistently yields a total of 10^4^ to 10^5^ viable primary MSCs per isolation at Passage 0. This robust yield provided an abundant cellular mass, allowing for the comprehensive acquisition of thousands of single-cell events during flow cytometry verification while ensuring more than sufficient high-quality total RNA for all downstream transcriptomic and molecular analyses.

#### 2.2.5. RNA Isolation and cDNA Synthesis

Total RNA was isolated from UC-MSCs cells using a modified phenol-chloroform method according to Chomczyński and Sacchi [11,48]. The purity and concentration of the obtained RNA were assessed spectrophotometrically. Approximately 1 µg of total RNA was used for complementary DNA (cDNA) synthesis. Reverse transcription was performed according to the manufacturer’s protocol using the High-Capacity cDNA Reverse Transcription Kit with RNase inhibitor (Applied Biosystems, Foster City, CA, USA). The obtained cDNA material was stored at −20 °C until further analysis. The methods have been described in detail using a reagent kit and laboratory equipment as described in a previous study [2,11,12,13,41,49,50,51].

#### 2.2.6. Transcriptomic Profiling (Microarray)

The GeneChip Human Gene 2.0 ST Array microarray system (Affymetrix, Santa Clara, CA, USA) was used to conduct high-throughput gene expression screening, allowing for the analysis of 48,226 genes. Transcriptomic profiling was performed on six arrays, using two biological replicates (*n* = 2) for each of three experimental groups defined by cord blood pH:

Acid group: pH < 7.35

Physiological group: pH 7.35–7.39

Alkaline group: pH ≥ 7.4

The microarray screen was used solely for exploratory and hypothesis-generating purposes. Given the descriptive nature and specific cohort size of this discovery phase (*n* = 2 per pH group), all initial transcriptome signals were considered provisional and subject to mandatory independent confirmation by targeted PCR in a larger validation cohort (*n* = 50). A detailed protocol for hybridization preparation and array scanning is described in a previous publication [8].

#### 2.2.7. Gene Expression Validation by qPCR

Validation of the microarray results was performed using quantitative real-time PCR (qPCR) on a StepOne Plus system (Applied Biosystems, Waltham, MA, USA). The expression of a panel of 10 genes was analyzed: *EGF*, *FGF2*, *FGFR1*, *FGFR3*, *GDNF*, *HGF*, *IGF1*, *NES*, *NGF* and *PGF.* Commercially available TaqMan probes (Applied Biosystems/Thermo Fisher Scientific) with the following catalog numbers were used for the determinations: *IGF1*: Hs00153126_m1, *HGF:* Hs00300159_m1, *EGF*: Hs0199990_m1, *GDNF*: Hs00181185_m1, *NGF*: Hs01113193_m1, *FGFR1*: Hs00241111_m1, *FGFR3*: Hs00179829_m1, *FGF2:* Hs00266645_m1, *NES*: Hs04187831_g1, *PGF*: Hs00182176_m1, *GAPDH:* Hs99999905_m1 (as an endogenous control). Gene expression levels (Relative Quantification, RQ) were calculated according to the 2^−DeltaDeltaCt^ method [52]. Glyceraldehyde-3-phosphate dehydrogenase (*GAPDH*) was used as a reference gene (internal control). Details of the procedure, reagents, and laboratory equipment were described in a previous work [2,11,12,13,41,49,50,51].

#### 2.2.8. Differential Gene Expression Analysis (DEG)

Identification of differentially expressed genes (DEGs) was performed using Transcriptome Analysis Console (TAC) software version 4.0.2 (Affymetrix/Thermo Fisher Scientific, Santa Clara, CA, USA) using Empirical Bayes ANOVA. The Bayesian model significantly increased the stability of variance estimates, which was methodologically crucial due to the limited number of biological replicates in the microarray groups (*n* = 2 per pH category). To clearly clarify the hierarchy of transcript selection for further validation steps, a nominal (uncorrected) significance threshold of *p* < 0.05 was applied at the single-gene level, combined with a strict biological cutoff of an absolute fold change greater than 2 (FC > 2, corresponding to a log_2_ FC > 1 threshold). Given the exploratory framework of this initial discovery step, individual FDR (False Discovery Rate) values generated by the TAC software were treated as strictly descriptive metrics. Enforcing a stringent FDR filter directly at the single-gene level at this stage would drastically reduce the statistical power of the assays and introduce an unacceptable risk of discarding potentially biologically relevant targets (Type II error/false negatives) that were successfully validated in the next step by targeted qPCR analysis.

#### 2.2.9. Functional Enrichment Analysis

Next, a multifaceted functional enrichment analysis based on signaling pathways (Pathway Analysis) and Gene Ontology (GO) was performed for the selected gene lists using g:Profiler [53]. To rigorously control for the effect of multiple testing and prevent false-positive functional assignments inherent in large-scale screening studies, a multiple testing correction was strictly enforced at the pathway level. The input query list (generated based on the previous nominal criteria of *p* < 0.05 and FC > 2) was subjected to rigorous statistical evaluation, in which the significance of enrichment of individual functional categories was verified based on the adjusted *p*-value (*p*_adj_). A strict false discovery rate (FDR) control threshold with a cutoff value < 0.25 was directly applied. Only pathways and biological processes that passed this adjusted significance barrier were considered statistically robust and included in the final dataset. Importantly, the analysis was performed multi-pronged: separately for up- and down-regulated genes, as well as for the global effect (all DEGs), allowing for precise and nuanced identification of biological processes modulated by pH changes in the UC-MSC microenvironment.

#### 2.2.10. Statistical Analysis

Statistical analysis of the collected data was performed using Statistica 13 software. The normal distribution of Quantitative variables was verified using the Shapiro–Wilk test. Differences between group means were assessed using the parametric Student’s *t*-test. Relationships between the analyzed parameters were evaluated using Pearson’s correlation coefficient. Gene expression data were presented on a logarithmic scale as log_10_RQ. A *p*-value < 0.05 was considered statistically significant.

Furthermore, due to the exploratory nature of the extended clinical correlations and the small sample sizes of some clinical subgroups, results with nominal *p* values ranging from 0.05 to 0.08 were interpreted and presented as statistical trends. However, we explicitly acknowledge that conducting multiple parallel comparisons in the correlation matrix increases the mathematical risk of false-positive results (Type I error). Therefore, all results falling within this “trend” range (0.05 = *p* < 0.08) are formulated with extreme caution and serve only as preliminary, hypothesis-generating indicators that require future validation in larger, independent cohorts.

## 3. Results

### 3.1. Confirmation of the Presence of Mesenchymal Stromal Cells

Microscopic analysis revealed the presence of spindle-shaped, mononuclear cells (Figure 1). Additionally, flow cytometry demonstrated the presence of the surface antigens CD73, CD90, CD105, and CD146, with the absence of CD45, CD31, CD19, and CD14, which constitutes an immunophenotype characteristic of MSCs (Figure 2a,b).

The present study is based on a two-step analytical strategy combining high-throughput transcriptomic profiling with targeted molecular validation in a larger clinical cohort. This approach allowed firstly for the unbiased identification of global mechanisms of cellular adaptation to pH changes and then for the precise assessment of the expression of key mediators of regenerative processes in the context of cord blood gas parameters.

### 3.2. Microarray Analysis

Using a high-throughput microarray approach, an initial screening of 48,226 genes was performed in a discovery subset of six individuals. This discovery phase was strictly exploratory, aiming to provide an unbiased direction for further molecular analyses. Therefore, the presented microarray dataset should be interpreted solely as a hypothesis-generating tool, and final biological significance can only be established after rigorous qPCR validation in an expanded clinical trial cohort (N = 50).

#### 3.2.1. Quantitative DEG Analysis

**Figure 3:**
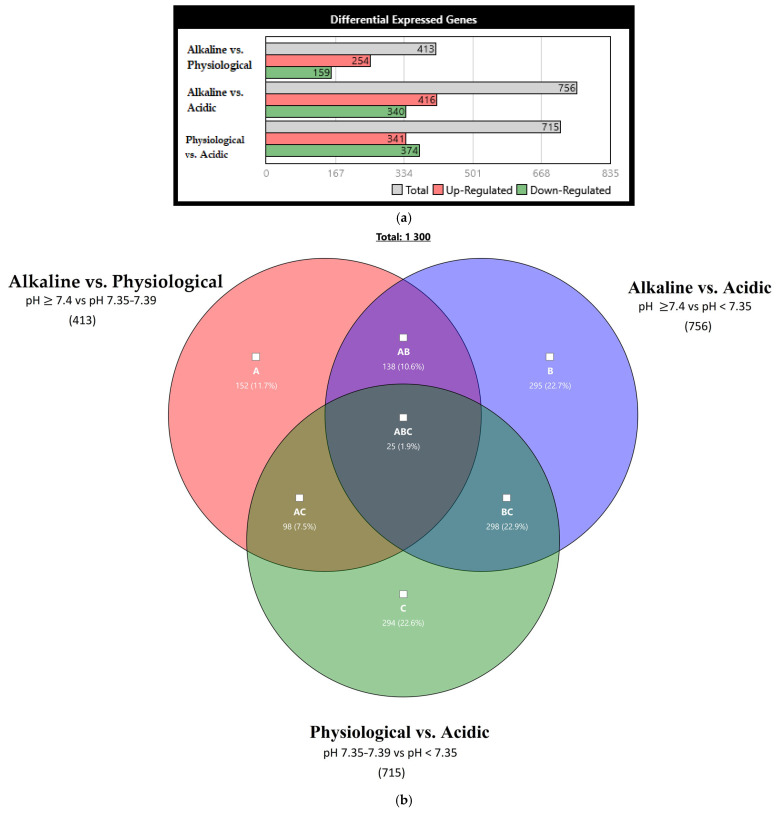
(**a**) Summary of the number of differentially expressed genes (DEGs) in UC-MSCs cells for individual experimental comparisons. (**b**) Venn diagram showing the relationships between sets of differentially expressed genes across pH gradients.

To quantify the impact of pH on the UC-MSCs transcriptome, a comparative analysis of differentially expressed genes (DEGs) was conducted across the three experimental ranges. The magnitude of transcriptional modulation followed a clear hierarchy depending on the pH gradient. Initial analysis of the transcriptomic response was performed using a nominal significance threshold of *p* < 0.05 and a fold-change |FC| > 2. This approach allowed for a comprehensive assessment of the gene expression changes across the pH gradient. Under these criteria, the most extensive changes were identified between acidic (pH < 7.35) and slightly alkaline (pH ≥ 7.4) conditions, involving a total of 756 DEGs (416 up-regulated and 340 down-regulated). A similarly high impact was observed when comparing the acidic group with the physiological range (pH 7.35–7.39), resulting in 715 DEGs. In contrast, the comparison between the physiological and slightly alkaline ranges yielded the lowest number of transcriptomic alterations (413 genes), with 254 genes showing increased expression in the alkaline group (Figure 3a).

Integration of these data sets via Venn analysis revealed that while each pH shift induces a highly specific response, there is a substantial overlap (298 genes) between the two comparisons involving acidic conditions. This suggests a consistent transcriptional “acidosis signature.” Furthermore, a core set of 25 genes (1.9% of all identified DEGs) was found to be consistently regulated across all pH transitions, representing the fundamental pH-sensitive core of the UC-MSCs transcriptome (Figure 3b).

The graph shows the total number of genes (gray) whose expression was significantly changed, divided into up-regulated (red) and down-regulated (green) genes. The analysis was performed using a statistical significance threshold of *p* < 0.05 (ebayes ANOVA method) and an expression coefficient of change |FC| > 2. The highest number of transcriptional changes (756 genes) was observed when comparing physiological conditions (pH ≥ 7.4) with acidotic conditions (pH < 7.35), suggesting a strong cellular response of UC-MSCs to a reduced pH environment (Figure 3a).

Global cross-referencing of the differentially expressed gene (DEG) lists using a three-dimensional Venn diagram configuration (FC > 2 *p* < 0.05, Empirical Bayes ANOVA) showed that the transcriptional profile of UC-MSCs is significantly shaped by the cord blood pH gradient (Figure 3a). To provide an immediate understanding of these transcriptome intersections, specific datasets were defined based on their directional contrasts: Comparison A (red circle) depicts the molecular contrast between the alkaline and physiological groups (pH ≥ 7.4 versus pH 7.35–7.39), Comparison B (blue circle) depicts the contrast between alkaline and acidic environments (pH ≥ 7.4 versus pH < 7.35), and Comparison C (green circle) tracks the physiological reference range versus the acidic group (pH 7.35–7.39 versus pH < 7.35). The largest absolute set of altered transcripts (756 DEGs) was identified in the boundary condition comparison (Comparison B), which also yielded the largest number of strictly unique expression changes (295 genes), highlighting the highly specialized, non-overlapping transcriptional response triggered by severe acidotic stress. Of particular note is the strong molecular correlation between the two low-pH axes (comparisons B and C), which share a massive intersection of 298 co-regulated genes (area BC). This common cohort represents nearly 40% of all acidification-induced transcriptome changes relative to the optimal physiological pH, strongly suggesting the activation of a stable, acid-dependent genetic signature responsible for structural adaptation and cell survival. In contrast, the core consensus response (area ABC) showed a relatively small pool of 25 genes (1.9% of all DEGs) consistently dysregulated across all three comparable subsets, indicating that although a small, universal multi-stress center exists, the overarching cellular response remains highly specific to a precise extracellular hydrogen ion concentration (Figure 3b).

#### 3.2.2. Functional Enrichment and Pathway Analysis

To transition from individual gene counts to robust biological insights, a more stringent statistical framework was applied for pathway enrichment analysis. To control for the risk of false-positive results inherent in large-scale functional testing, only biological processes and pathways with a False Discovery Rate (FDR) below 0.25 were considered significant.

This multi-level filtering strategy ensured that the identified functional themes—such as the marked transition from cell proliferation to extracellular matrix (ECM) remodeling in acidic conditions—represent the most consistent and biologically relevant signals within the dataset. While the nominal *p*-value highlights the overall magnitude of transcriptomic shifts, the FDR-adjusted pathway analysis focuses on the coordinated regulation of functionally related gene sets.

#### 3.2.3. Global Effect of Environmental pH on the UC-MSCs Transcriptome

To evaluate the impact of pH variations on the functional state of umbilical cord-derived MSCs (UC-MSCs), a global gene expression profile analysis was performed using Affymetrix HuGene2.0 microarray across three experimental groups: acidic (pH < 7.35), physiological (pH = 7.35–7.39), and slightly alkaline (pH ≥ 7.4).The global analysis (FDR < 0.25) revealed significant modulation of critical signaling pathways, particularly those related to enzyme-coupled receptor activity (*p*_adj_ = 3.44 × 10^−12^) and signaling receptor binding (*p*_adj_ = 6.72 × 10^−4^). We identified key transcriptional changes within the fibroblast growth factor family (*FGF5*, *FGF7*, *FGF10*) and their corresponding receptors (*FGFR2*, *EPHA3*). Furthermore, pH variations significantly influenced extracellular matrix (ECM) organization (*p*_adj_ = 1.91 × 10^−5^) and cell adhesion processes, with genes such as *COMP*, *DCN*, *LUM*, and *VEGFA* playing central roles. These findings demonstrate that environmental pH is a critical regulator of cell–matrix interactions and the angiogenic potential of UC-MSCs at the molecular level (Supplementary Table S1). The effect of pH on the angiogenic potential of UC-MSCs should also be confirmed in functional tests.

#### 3.2.4. Effect of Environmental Acidification (pH < 7.35) on the MSCs Phenotype

Comparison of the acidic group with the physiological pH group revealed the presence of 176 genes with increased expression (log_2FC > 1) and 241 genes with decreased expression (log_2FC < −1). Overexpressed genes: Functional enrichment analysis revealed strong activation of pathways related to extracellular matrix organization (*p*_adj_ = 1.36 × 10^−2^) and cell adhesion (*p*_adj_ = 1.24 × 10^−9^), with a significant contribution from *FMOD*, *DPT*, *COL15A1*, and *COMP*. Enrichment of actin cytoskeleton and actomyosin structure reorganization was also observed. A significant change was the activation of the canonical Wnt signaling pathway (*GPC3*, *FGF10*, *DKK2*) and TGF-beta receptor binding (*TGFB3*, *SMAD6*) (Supplementary Table S2).

Downregulated genes: Under conditions of reduced pH, a drastic inhibition of cell cycle and proliferation-related processes was observed. The most significant categories included nuclear division (*p*_adj_ = 3.33 × 10^−8^), cell division (*p*_adj_ = 8.93 × 10^−4^), and cell population proliferation (*p*_adj_ = 1.28 × 10^−2^). This phenomenon was correlated with downregulation of key cell cycle regulators, such as *CDK1*, *CDC20*, *AURKA*, and *TOP2A* (Supplementary Table S3).

#### 3.2.5. Characterization of MSCs in Alkaline Conditions (pH ≥ 7.4)

Comparison of the physiological pH group with the alkaline group revealed a more subtle but biologically significant profile of changes. In the higher pH group, overexpression of genes associated with the immune response was observed, including the TLR3 signaling pathway (*TLR3* gene, *p*_adj_ = 4.99 × 10^−2^) and interferon-related genes. Genes with decreased expression at pH ≥ 7.4 were primarily associated with structural matrix components (e.g., *MMP1*, *FMOD*, *DPT*), indicating a lower intensity of ECM remodeling compared to physiological and acidic conditions (Supplementary Table S4).

#### 3.2.6. Comparison of pH Extremes: Acidic vs. Alkaline Environments

Direct comparison of the acidic group (pH < 7.35) with the alkaline group (pH ≥ 7.4) confirmed the dominant effect of low pH on promoting the migratory and structural phenotypes at the expense of the proliferative one.

Functional enrichment analysis of genes overexpressed at low pH indicated a dominant role for pathways related to receptor signaling and structural remodeling. The highest significance level was observed for the enzyme-linked receptor protein signaling pathway (*p*_adj_ = 8.76 × 10^−11^), represented by genes such as *FGF10*, *FGFR2*, *TGFB3*, *BAMBI*, and *GPC3*. In parallel, a strong activation of processes related to extracellular matrix (ECM) organization and cell adhesion (*p*_adj_ = 1.82 × 10^−5^) was observed, as confirmed by the high expression of structural genes: *FMOD*, *DCN*, *LUM*, *COL15A1*, and *DPT.* These results suggest that low pH induces a phenotype in UC-MSCs that is directed towards intensive modification of the tissue environment and increased mobility, which is further supported by the enrichment of the cell migration pathway (*p*_adj_ = 4.86 × 10^−3^; *SORBS1*, *IQGAP2* genes) (Supplementary Table S5). In the group of genes with reduced expression in an acidic environment, processes related to cell–substrate interactions and cell surface homeostasis dominated. Statistically significant silencing of collagen binding pathways (*p*_adj_ = 1.65 × 10^−3^) and glycosaminoglycan (*LUM*, *PRELP*) was identified. Importantly, although the UP gene analysis noted the presence of growth pathways, the DOWN gene analysis revealed a simultaneous reduction in the expression of another set of genes responsible for positive regulation of cell population proliferation (*p*_adj_ = 4.96 × 10^−3^), including *FGF10* and *TGFB3* (indicating their complex, bidirectional regulatory role). Cell surface components (*p*_adj_ = 2.62 × 10^−3^) and surface receptor signaling pathways were also silenced (Supplementary Table S6).

This initial identification allowed the identification of primary sets of genes sensitive to environmental changes (Table 3). Although these transcriptome variations indicate operable genetic pathways, they should be interpreted cautiously as indicators generating hypotheses about the in vitro cell state, rather than as direct evidence of established functional or proteomic perturbations.

### 3.3. Results qPCR Validation and Clinical Correlations

#### 3.3.1. Influence of Blood Gas Parameters on Growth Factor Expression

To confirm the observations from the transcriptomic analysis and assess their clinical significance, qPCR analysis was performed on an expanded study group (n = 50). The focus was on the expression of key growth factors and their receptors in the context of cord blood gas parameters and the neonatal clinical status (Table 4).

#### 3.3.2. Correlation of Gene Expression with Cord Blood pH and pCO_2_

**Figure 4:**
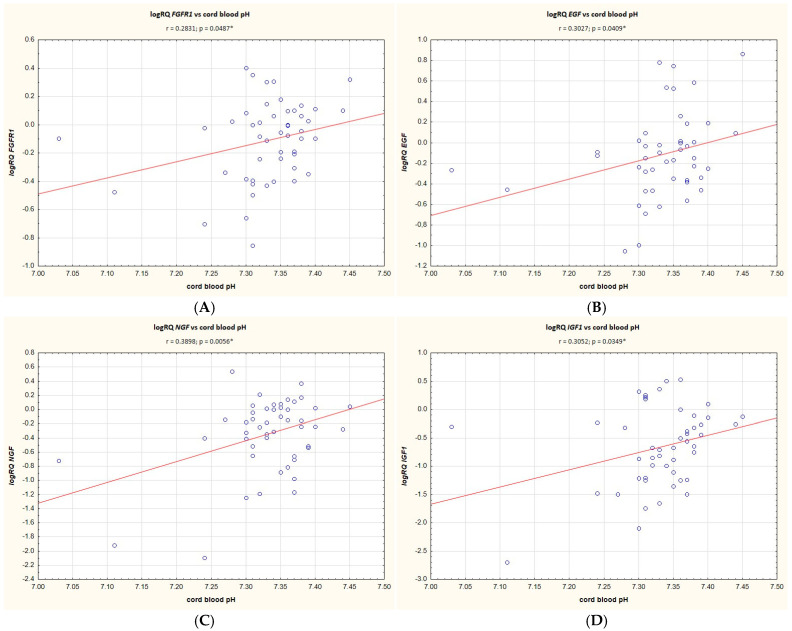
(**A**). Relationship between logRQ *FGFR1* in UC-MSCs and umbilical cord blood pH (r = 0.2831; *p* = 0.0487). (**B**). Relationship between logRQ *EGF* in UC-MSCs and umbilical cord blood pH (r = 0.3027; *p* = 0.0409). (**C**). Relationship between logRQ *NGF* in UC-MSCs and umbilical cord blood pH (r = 0.3898; *p* = 0.0056). (**D**). Relationship between logRQ *IGF1* in UC-MSCs and umbilical cord blood pH (r = 0.3052; *p* = 0.0349). Axis readouts for gene expression are presented as relative quantification values subjected to common logarithmic transformation (log_10_RQ). * *p* < 0.05.

**Figure 5:**
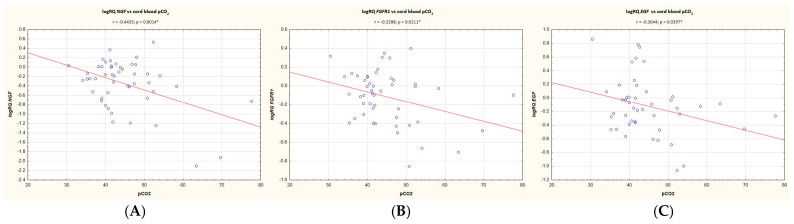
(**A**). Relationship between logRQ *NGF* in UC-MSCs and umbilical cord blood pCO_2_ (r = −0.4435; *p* = 0.0014). (**B**). Relationship between logRQ *FGFR1* in UC-MSCs and umbilical cord blood pCO_2_ (r = −0.3288; *p* = 0211). (**C**). Relationship between logRQ *EGF* in UC-MSCs and umbilical cord blood pCO_2_ (r = −0.3044; *p* = 0.0397). Axis readouts for gene expression are presented as relative quantification values subjected to common logarithmic transformation (log_10_RQ). * *p* < 0.05.

The qPCR results demonstrated high consistency with microarray data, particularly in the area of signaling pathways associated with receptors with kinase activity. A statistically significant positive correlation was found between cord blood pH and the expression of genes crucial for cell proliferation and survival: *FGFR1* (r = 0.2831), *EGF* (r = 0.3027), *NGF* (r = 0.3898), and *IGF1* (r = 0.3052) (Figure 4, Table 5). This relationship was further confirmed by the results regarding the partial pressure of carbon dioxide (pCO_2_), which is the main determinant of respiratory acidity. A negative correlation was demonstrated between pCO_2_ level and the expression of *NGF* (r = −0.4435), *FGFR1* (r = −0.3288), and *EGF* (r = −0.3044) (Figure 5, Table 5). The lack of correlation with pO_2_ and bicarbonate concentration (cHCO_3_) suggests that pH and hypercapnia, not oxygen deficiency per se, are the primary regulators of the expression of these factors in UC-MSCs.

#### 3.3.3. The Impact of Threatened Neonatal Asphyxia on the Regenerative Potential of UC-MSCs

Analysis taking into account the neonatal clinical condition revealed that threatened asphyxia—a condition closely associated with metabolic and respiratory acidosis—significantly reduced the expression of the studied growth factors. Significantly lower transcript levels of *EGF* (*p* = 0.009), *FGFR1* (*p* = 0.047), and *NGF* (*p* = 0.042) genes were observed in these groups. At the same time, marginal statistical trends towards downregulation were noted for *GDNF* (*p* = 0.071) and *IGF1* (*p* = 0.079); however, due to the high risk of false-positive inflation in multiple testing, these speculative trends are interpreted strictly as preliminary observations.

#### 3.3.4. Correlations Between the Expression of Individual Growth Factor Genes in UC-MSCs

**Figure 6:**
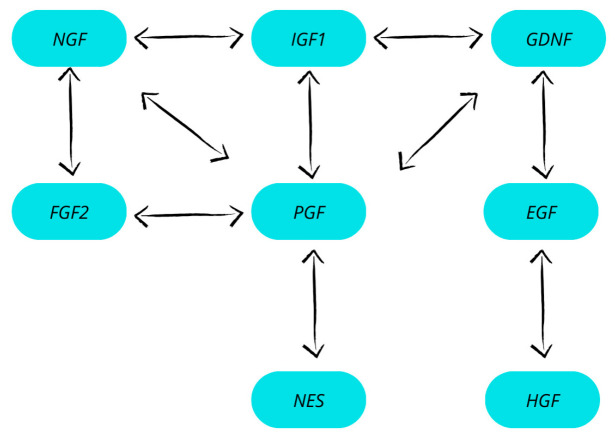
Positive correlations between the expression of growth factors in UC-MSCs. Network of positive correlations between growth factor expression levels. Arrowed lines (bidirectional) indicate statistically significant positive correlations between pairs of growth factors. Line thickness/connectivity reflects the associations observed in the statistical analysis.

Statistically significant positive correlations were observed between the expression of individual genes (Figure 6). The identified relationships are presented in Table 5. The presented correlations apply to the entire study group. The data indicate the existence of a network of relationships between individual growth factors. Our results showed that NGF positively correlates with IGF1 (r = 0.357), PGF (r = 0.404), and FGF2 (r = 0.300). IGF1 also positively correlates with GDNF (r = 0.312) and PGF (r = 0.362). GDNF correlates with PGF (r = 0.543) and EGF (r = 0.446). EGF, in turn, also correlates with HGF (r = 0.543). Furthermore, we demonstrated a correlation between PGF expression and FGF2 (r = 0.451) and NES (r = 0.294).

Analysis of the correlation network (Figure 6) and associated statistical matrices (Table 5) allows for the biological interpretation of UC-MSCs cell behavior under specific microenvironmental conditions, such as acidosis and hypercapnia induced by threatened perinatal hypoxia. The presented network is not a random combination of variables but reflects a coordinated, functional transcriptional response program to chemical stress. Under physiological pH conditions, the expression of the studied growth factors remains in a state of homeostasis, safeguarding basic cell proliferative processes. The situation changes under conditions of acidosis (pH < 7.35) and increased pCO_2_. Strong, positive correlations within the network (centered around central signaling nodes such as *GDNF* and *NGF*) indicate that UC-MSCs, under metabolic stress, switch their expression profile from classical mitogenic pathways (associated with intense cell division, represented by silenced *EGF* and *FGFR1* axes) to an integrated rescue and neuroprotective phenotype. The biological significance of this co-regulation lies in the fact that cells—in the face of unfavorable blood gas conditions—dynamically adapt, increasing transcription of factors responsible for tissue survival, extracellular matrix remodeling, and protection of neuronal lineages (stable *GDNF* expression). The high network coherence demonstrated in mathematical models demonstrates that changes in the perinatal microenvironment can act as a global molecular switch, triggering a cascading, synergistic paracrine response, which is fundamental for assessing the therapeutic potential of cells in regenerative medicine.

Our results suggest that increasing the expression of even one of the indicated growth factors can trigger a cascade of subsequent changes. This may indicate the activation of complex mechanisms designed to adapt the cell to the prevailing microenvironmental conditions. However, it remains to be seen whether the same relationships occur at the protein level.

#### 3.3.5. Extended Correlation Analysis: The Influence of Clinical and Hematological Parameters on the Expression Profile of UC-MSCs

In addition to the influence of acid–base balance, the influence of other clinical and hematological variables on the transcriptional potential of UC-MSCs was analyzed. These results shed new light on the factors determining the molecular profile of these cells.

Auxological parameters and neonatal maturity

The analysis revealed a significant correlation between neonatal birth weight and the expression of key angiogenic and growth factors. Significantly higher expression of *FGF2* (*p* = 0.046) and *PGF* (*p* = 0.036) was observed in the group of neonates weighing > 3500 g compared to the group with a lower birth weight. Moreover, neonatal maturity measured by gestational age (GA) correlated negatively with nestin expression (*NES*) (r = −0.344; *p* < 0.05), which may suggest a gradual silencing of the neural progenitor cell profile with increasing gestational age (Table 4 and Table 5).

Obstetric history and morphological parameters

Analysis of the order of pregnancy and delivery yielded interesting results. A negative correlation was found between the number of pregnancies/deliveries and the expression of the *FGFR1* receptor (r = −0.34; *p* < 0.05) and the *HGF* factor (r = −0.28 for delivery; *p* < 0.05) (Table 4 and Table 5). This suggests that maternal obstetric status may subtly modulate the basal regenerative potential of cord cells.

In the area of hematological parameters, the correlation between white blood cell (WBC) counts and growth profiles is particularly noteworthy. Higher WBC count (11–15 10^9^/L) was associated with significantly lower expression of *EGF* (*p* = 0.012) and *NES* (*p* = 0.014). Additionally, higher hemoglobin concentration (>11.5 g/dL) and higher MCHC index corresponded with increased expression of *NES* and *NGF*, indicating a link between red blood cell status and neurotrophic potential of cells (Table 4 and Table 5).

### 3.4. Summary of Results

The selection of genes for qPCR analysis was not accidental, but rather a logical continuation and refinement of the results obtained during the microarray profiling phase. Global enrichment analysis (g:Profiler) clearly indicated that an acidic environment (pH < 7.35) induces a drastic downregulation of pathways associated with cell proliferation (GO:0008283) and enzyme-coupled receptor signaling (GO:0007167), while simultaneously reducing the expression of key receptors such as *FGFR2.* To verify these observations in a broader population (n = 50), genes acting as “hubs” in the identified pathways were selected for qPCR validation: *FGFR1*: as a key FGF family receptor, complementing the picture of changes in *FGF/FGFR* signaling observed in microarray. *EGF*, *IGF1*, and *NGF* are fundamental ligands driving the cell cycle and growth processes, whose inhibition under acidotic conditions directly explains the decrease in cell mitotic activity observed in microarray. Correlative analysis with umbilical cord blood gas parameters (pH, pCO_2_) and clinical status (imminent asphyxia) allowed for the translation of these in vitro observations into the clinical setting. The qPCR results provide confirmation of the transcriptomic data—the demonstrated positive correlation of *FGFR1*, *EGF*, *IGF1*, and *NGF* expression with blood pH confirms that metabolic and respiratory acidosis are the main factors inhibiting the paracrine and proliferative potential of UC-MSCs. Thus, targeted analysis (qPCR) refined the global signal from the microarray, identifying specific molecular axes (e.g., the *EGF–FGFR* axis) that are attenuated as a result of unfavorable perinatal conditions (Table 6). Due to the use of two methods, our results are characterized by high reliability at the molecular level, however, for a complete picture, the obtained conclusions should be confirmed at the protein level and in functional tests.

## 4. Discussion

### 4.1. Influence of Blood Gasometric Parameters on the Regenerative Potential of UC-MSCs

The obtained results of microarray analysis clearly indicate that environmental pH is a critical factor modifying the gene expression profile in umbilical cord mesenchymal stromal cells (UC-MSCs). The highest number of differentially expressed genes identified in comparisons of extreme pH values suggests that these cells possess high metabolic sensitivity to changes in acidity, responding with profound transcriptional reprogramming. A key finding from the quantitative analysis is the existence of the so-called “acidosis signature”—a group of nearly 300 genes that respond consistently to lower pH, regardless of whether the reference point is the physiological or slightly alkaline range. This suggests that UC-MSCs possess an evolutionarily conserved response mechanism to acidotic stress that predominates over more subtle adaptive changes at higher pH ranges. One of the most striking effects of lowering pH below 7.35 is the drastic silencing of the cell cycle machinery. Strong downregulation of genes such as *CDK1*, *AURKA*, *BUB1*, and *TOP2A* indicates inhibition of cell progression through subsequent phases of mitosis. From a biological perspective, this can be interpreted as a protective mechanism. In the face of unfavorable metabolic conditions (low pH, often accompanying hypoxia), stromal cells enter a state of limited activity (quiescence). This reduction in the rate of division allows for the conservation of energy resources and minimization of DNA damage, which is crucial for maintaining the integrity of the stromal cell pool in the tissue niches of the umbilical cord. While proliferative processes are silenced, UC-MSCs in an acidotic environment demonstrate a dramatic increase in the activity of genes related to the extracellular matrix (ECM) and adhesion. Overexpression of proteoglycans and collagens (e.g., *FMOD*, *DCN*, *LUM*, *COL15A1*) with a concomitant decrease in the expression of metalloproteinases (e.g., *MMP1*) suggests a shift in cellular strategy from expansion to structure and stabilization. Pathway enrichment analysis revealed activation of signaling cascades associated with enzymatic receptors, including the Wnt and TGF-beta pathways. These pathways are crucial for tissue regeneration and angiogenesis. The role of genes such as *FGF10* and *TGFB3* is particularly interesting. Their overexpression in acidotic conditions, despite a general inhibition of proliferation, indicates that UC-MSCs subjected to pH stress may paradoxically acquire a higher paracrine potential. This may indicate that cells isolated from cord blood with a low pH (e.g., from acidotic deliveries) demonstrate a stronger tendency to induce repair processes in recipient tissues, which has significant implications for regenerative medicine. In summary, at physiological and alkaline pH, cells are oriented towards immunological homeostasis and moderate metabolic activity. At acidic pH (<7.35) there is a shift towards a migratory–structural phenotype, characterized by inhibition of division but enhanced interaction with the matrix and activation of regenerative pathways.

The key finding of this study is the demonstration in qPCR analysis that the expression profile of growth factors in UC-MSCs is closely modulated by the acid–base balance of umbilical cord blood. The observed positive correlation between pH and the expression of *NGF*, *EGF*, *FGFR1*, and *IGF1* (and a negative correlation with pCO_2_) provides a mechanistic explanation for the results of microarray analysis, in which acidosis (pH < 7.35) induced a drastic silencing of cell cycle and proliferation pathways (including ↓*CDK1*, *AURKA*). Our data suggest that the inhibition of cell division results directly from the deficiency of key mitogens and their receptors induced by low pH.

Integration with previous studies on the pH microenvironment: The present results represent an important continuation of our earlier work, which systematically indicates the key role of cord blood biochemical parameters in programming the UC-MSCs phenotype [2,11,12,13]. We have previously demonstrated that an acidic environment induces specific adaptive and compensatory mechanisms in these cells. In studies of pluripotency and survival markers, we observed that lower cord blood pH correlates with higher expression of the SOX2 factor and genes from the apoptosis inhibitor family (*BIRC2*, *BIRC3*, *BIRC5*) [12,13]. This suggests that in response to acid stress, these cells enter a state of increased resistance to programmed cell death, retaining a more “primordial” character. This is consistent with the current microarray results, indicating a shift in cell survival and intensive structural remodeling at the expense of active proliferation. In parallel, our work on immunomodulatory and proangiogenic potential confirmed that pH and pCO_2_ significantly modulate the secretion of the anti-inflammatory protein TSG-6 and interleukins (IL-2, IL-6) [2]. We also demonstrated that hypoxic states (resulting from, among others, hypertension or maternal hypothyroidism) lead to a compensatory increase in the expression of VEGF family factors [11]. Integrating these data with the current discovery of suppression of the *NGF/EGF/IGF1* axis allows for the formulation of a comprehensive model of the UC-MSCs response to acidosis: Decreased expression of classical mitogens (*EGF*, *IGF1*, *FGFR1*) inhibits the cell cycle. Increased expression of antiapoptotic genes (IAPs), pluripotency factors (*SOX2*), and mediators of regeneration and angiogenesis (*VEGF*, *TSG-6*, *GDNF*).

Based on the integration of global transcriptome screening and targeted qPCR validation, we propose a comprehensive biological model of UC-MSCs cell adaptation to the perinatal microenvironment. Unfavorable blood gas conditions (acidosis and hypercapnia) inhibit classical mitogenic axes (*EGF*, *IGF1*, *FGFR1*), which may suggest cell exit from the active cell cycle. Simultaneously, a compensatory survival phenotype is activated, characterized by intense extracellular matrix remodeling, increased migratory potential, and sustained neuroprotective properties through stable *GDNF* gene expression. However, despite the statistical significance of the presented relationships, qPCR analyses are correlational in nature and do not allow for direct determination of cause-and-effect relationships. Because functional parameters or protein secretory levels were not directly assessed in this study, the observed changes at the mRNA level should not be interpreted as permanent or irreversible functional impairment of isolated UC-MSC cells, but rather as transient transcriptional adaptation to perinatal chemical stressors. To fully verify the proposed model, it is necessary to extend future analyses to include assessment of trophic factor concentrations at the protein level (e.g., using ELISA) and to perform functional tests to verify the actual capacity of cells to proliferate, migrate, and paracrinely stimulate angiogenesis.

#### Molecular Analysis of Growth Factors in the Context of the Literature

Observations regarding *NGF* at the molecular level are complementary to the reports by Huang et al., indicating destabilization of the NGF protein upon exposure to low pH [30]. Bray et al., in turn, observed that acidosis can activate NGF receptors in sensory neurons, increasing their sensitivity, which is important in the pathophysiology of pain [54]. We observed a similar relationship for EGF and IGF1—the literature confirms their reduced activity in acidosis, demonstrating stronger activation of EGFR at alkaline pH and inactivation of this receptor under hypercapnic conditions [55,56]. This correlates with clinical findings demonstrating decreased serum IGF-1 concentrations in the course of metabolic acidosis [57]. Importantly, the effect of pH on FGFR1 expression we demonstrated is a new observation, not previously described in the literature. GDNF behaves differently, with its expression increasing in UC-MSCs under low pH conditions. This may be due to its involvement in long-term adaptation; unlike NGF, which responds dynamically and briefly to acute conditions, elevated GDNF levels persist for a longer period [20]. Functional remodeling and therapeutic implications: The effect of acidosis on reduced proliferation of smooth muscle cells, endothelial cells, and lymphocytes has already been described [58,59,60]. Our results suggest that the mechanism of this phenomenon in UC-MSCs is directly related to the suppression of *NGF*, *EGF*, *FGFR1*, and *IGF1* genes, which in the case of endothelial cells additionally results in inhibition of angiogenesis. An integrated summary of transcriptomic changes indicates profound functional remodeling of UC-MSCs. While acidosis stimulates matrix organization and migration processes, it simultaneously impairs the cells’ ability to bind collagen and changes their surface architecture. This dual effect suggests that low pH shifts UC-MSCs from a mode of stable adhesion and physiological proliferation to a mode of rescue structural remodeling and migration. Consequently, UC-MSCs originating from an unfavorable microenvironment are not damaged cells but functionally adapted to stress conditions, which determines their subsequent paracrine and therapeutic activity ex vivo. Unfavorable blood gas parameters may significantly limit the cells’ potential to stimulate growth, while maintaining the long-term adaptive response mediated by GDNF.

### 4.2. Threatened Neonatal Asphyxia and the Neuroprotective Profile of UC-MSCs

Imminent neonatal asphyxia, manifested by hypoxia and impaired gas exchange, is a critical factor determining the transcriptional profile of UC-MSCs. This condition leads to profound acid–base disturbances, which are a key diagnostic criterion for asphyxia [61,62]. The downward trend in *EGF*, *IGF1*, and *FGFR1* gene expression observed in this group is directly correlated with their sensitivity to metabolic acidosis. IGF1 has been attributed a particular role in fetal protection, its crucial importance for embryonic survival confirmed by studies in mouse models (Igf-1 knockout leading to lethality) [25]. The suppression of IGF1 observed in conditions of asphyxia and low pH suggests a weakening of this protective barrier in situations of extreme perinatal stress. In contrast to the above factors, NGF exhibits a unique, biphasic response dynamics. Despite its overall positive correlation with pH, an increase in its expression was observed in cases of acute asphyxia, which we interpret as the activation of a compensatory mechanism. *NGF* plays a fundamental role in promoting neuronal maturation and neuroprotection in response to injury [63], and its increase in acute conditions likely aims to minimize neurological losses resulting from hypoxia. However, our studies indicate a threshold to this mechanism—in conditions of persistent acidosis (pH < 7.30), NGF levels rapidly decline. This phenomenon can be explained by the nature of NGF as a neurotrophin that responds to acute conditions in a dynamic but short-lived manner [20]. This suggests that UC-MSCs derived from neonates with asphyxia may exhibit transiently enhanced neuroprotective properties, which, however, disappear with prolonged acidosis. In conclusion, the cumulative decrease in *GDNF*, *EGF*, *IGF1*, and *FGFR1* expression during asphyxia and associated acidosis suggests significant functional impairment of UC-MSCs. These cells may be characterized by impaired proliferation, limited migration, and reduced angiogenic and differentiation potential, which should be taken into account when assessing their clinical utility.

It should be emphasized, however, that the presented results concern only the transcriptional profile of UC-MSC cells, which does not allow for a clear definition of their ultimate functional properties. The effect of threatened neonatal asphyxia on the expression of GDNF, EGF, IGF1, and FGFR1 genes requires independent confirmation at the protein level, as well as functional tests assessing the ability of MSCs to proliferate, migrate, and stimulate angiogenesis. The significantly lower abundance of transcripts of key growth factors observed in the subgroup of neonates with threatened hypoxia indicates early molecular reactivity of UC-MSC cells in response to severe, acute perinatal stress. However, given that these parameters were measured solely at the mRNA level, these results suggest only a transient downregulation of regenerative pathways in vitro and do not prove definitive or irreversible functional impairment of this clinical donor material.

### 4.3. The Impact of Clinical Parameters and Neonatal Maturity on the Phenotype of UC-MSCs

The significant positive correlation between neonatal birth weight and the expression of proangiogenic factors (*FGF2*, *PGF*) is highly consistent with the results of microarray analysis, which noted a significant enrichment of pathways related to circulatory system processes. This suggests that UC-MSCs “inherit” the fetal metabolic profile—higher body weight and the associated need for vascularization promote a stronger angiogenic phenotype of cells already during fetal life.

Another key factor modulating the UC-MSCs transcriptome is neonatal maturity, measured by gestational age (GA, HBD). The negative correlation we observed between HBD and nestin expression (*NES)* reflects the natural ontogeny of these cells. Nestin is a recognized marker of early neurogenic stem cells, and its presence indicates intensive development or ongoing tissue regeneration [64,65].

The decline in NES expression typical of embryonic development with progressive cell differentiation and age [65,66,67] was confirmed in our study at the molecular level of UC-MSCs. Importantly, higher NES expression in neonates with lower HBD may have significant clinical implications. Given the increased risk of cerebral palsy in premature infants [68], elevated nestin levels in their stem cells may represent an endogenous compensatory mechanism aimed at supporting repair processes in response to biological immaturity of tissues.

### 4.4. Synergy and Intergenic Interactions in UC-MSCs

The numerous correlations we demonstrated between the growth factors studied indicate the existence of an integrated signaling network in UC-MSCs. The decrease in paracrine activity for ECM adhesion and remodeling at low pH, observed in microarray, is reflected in the weakening of these molecular connections.

#### 4.4.1. FGFR, EGF, and IGF1 Axes: Mechanism of Proliferation

Our results confirm the key role of the FGFR axis in regulating the growth potential of UC-MSCs. The positive correlation found between *FGFR1*, *FGFR3*, and *IGF1* is consistent with reports of stimulation of IGF1 levels by FGFR signaling [18]. Similarly, the demonstrated association of *EGF* with *FGFR1* (but not with *FGF2/FGFR3)* is consistent with known interactions between EGFR and FGFR4 receptors and evidence for FGFR stimulation by activated EGFR [69,70]. Furthermore, the synergistic effects of *EGF* and *HGF*, manifested in our results by a strong correlation, are confirmed by functional studies, where the combination of these factors induces proliferation more strongly than either factor alone [71,72,73].

#### 4.4.2. Neuroprotection: NGF vs. GDNF

Despite their similar functions, the lack of correlation between *NGF* and *GDNF* in our studies supports the hypothesis of their independent activation: *NGF* in acute conditions, and *GDNF* in chronic conditions [20,74]. The positive correlation between *EGF* and *GDNF* may, in turn, explain the mechanism described by Wang et al., where the action of *GDNF* was dependent on the presence of *EGF* [75]. This suggests that in conditions of acidosis (chronic), *GDNF* can act independently, whereas in acute asphyxia, its activity requires synergy with *EGF.* We also demonstrated strong associations of *NGF* with the *FGF* family (*FGF2*, *FGFR1*, *FGFR3*). This is consistent with observations of *FGF2/NGF* synergy in neuronal differentiation and the induction of *FGFR* receptors by *NGF* [76,77,78]. These relationships, along with *GDNF*–*FGFR1/3* [79] and *FGFR1*–*HGF* [80] correlations, form a dense network supporting cell survival.

#### 4.4.3. Angiogenesis and Neurotrophic Potential

UC-MSCs exhibit a potentially neuroprotective profile due to the interdependence of *GDNF*, *NGF*, and *IGF1*. Their synergy, evident in our correlations, significantly increases neuronal survival and axonal branching [22,81]. Importantly, the proangiogenic factor *PGF* correlated with *GDNF*, *IGF1*, *NGF*, and *NES* (nestin). Although the literature rarely describes these relationships directly, the functional association of these factors with the formation of capillary structures and high expression of nestin in endothelial cells [26,78,82,83] confirms the involvement of UC-MSCs in neovascularization processes.

#### 4.4.4. Summary of Correlations

In summary, the identified gene interactions indicate the multifaceted effects of UC-MSCs. The consistency of our results with the literature (e.g., the lack of an *FGF2*–*EGF* correlation [77] and a statistically significant FGF2–PGF correlation, extending the observations of Martinez-Fierro [84]) demonstrates that UC-MSCs constitute a finely tuned paracrine tool, whose effectiveness, however, is closely dependent on the microenvironmental conditions.

Importantly, despite the robustness of the identified statistical associations, it is important to emphasize that our quantitative real-time polymerase chain reaction (qPCR) analyses remain correlational in nature. Therefore, the observed associations between cord blood pH, pCO_2_, and clinically threatened neonatal hypoxia and growth factor expression profiles in UC-MSCs do not allow for the establishment of direct molecular causality. These transcript-level changes represent initial transcriptional adaptations to environmental stress but do not definitively confirm parallel changes in secretome protein levels or ultimate cellular behaviors. Comprehensive functional and protein-level validation—such as enzyme-linked immunosorbent assays (ELISAs) for growth factor quantification, Western blotting for receptor activation, and phenotypic assays assessing proliferation, migration, and endothelial tubule formation—is critically necessary to establish causality before these parameters can be confidently used as selection criteria for therapeutic stromal cells.

## 5. Strengths and Limitations

A key strength of this study is its integrated methodological process, successfully combining objective, high-throughput microarray screening with targeted validation using qPCR. The large size of our validation cohort (N = 50) provides a solid statistical basis for drawing robust correlational conclusions in the perinatal clinical setting. Furthermore, to the best of our knowledge, this work represents the first such detailed mapping of the correlation of cord blood gas parameters directly with the comprehensive transcriptomic profile of primary UC-MSCs. These findings have significant implications for both regenerative medicine and clinical cord blood biobanking, demonstrating that baseline blood gas matrices (such as pH and CO_2_) can potentially serve as rapid, noninvasive proxies for biological programming of isolated stromal cells.

Importantly, special care must be taken when translating these findings into the context of clinical biobanking and donor tissue selection. Although cord blood gases and pH indicators show significant potential as early, objective screening markers of UC-MSCs transcriptomic binding, they cannot currently be used as definitive, stand-alone criteria for release or rejection. It must be clearly emphasized that robust, multicenter validation, strictly at the protein expression level (e.g., quantification of the cellular secretome) and through comprehensive functional testing (e.g., in vitro and in vivo therapeutic efficacy models assessing proliferation, migration, and angiogenesis), remains an absolute prerequisite. Until long-term functional stability and proteomic profiles are fully established, these perinatal parameters should be considered complementary, hypothesis-generating biomarkers rather than as routine operational standards for donor tissue qualification in stromal cell repositories.

We acknowledge that a major limitation of the present study is the relatively small sample size used in the initial, global transcriptome screen (N = 6), which included two biological replicates for each pH category. Although limited sample sizes are standard and widely accepted in high-throughput exploratory microarray designs due to high operational costs and screening constraints, these exploratory data should be interpreted with caution, as hypothesis-generating rather than definitive. To effectively mitigate this limitation and control the false discovery rate (FDR) inherent in small-scale profiling, we implemented a strict two-step analytical strategy. Exploratory microarray reads were subjected to multi-step statistical filtering (empirical Bayesian analysis of variance combined with strict control of FDR at the pathway level) and, crucially, were then validated by targeted quantitative real-time PCR (qPCR) in a significantly expanded, statistically robust clinical cohort (N = 50).

Furthermore, readers should exercise caution when interpreting the multiple correlation matrices presented in this document. Because the extended clinical and hematological correlation analyses involved a significant number of simultaneous statistical comparisons, the likelihood of encountering type I errors (false positives) is elevated. This is particularly important for reported statistical trends within the *p* = 0.05–*p* = 0.08 range, which should be interpreted strictly as preliminary biological trends rather than definitive, confirmatory associations.

Importantly, while our previous studies have identified isolated biochemical associations with stromal cell properties, this study provides the first unbiased, high-throughput, global transcriptomic model of human UC-MSCs in direct correlation with paired umbilical cord blood gas parameters and precise, computerized Dawes–Redman intrapartum cardiotocography (CTG/STV) parameters. The discovery of specific, microenvironment-dependent transcriptomic regulation of the *FGFR1* axis and its tightly coordinated network co-regulation with the *EGF/NGF/IGF1* pathways has never been documented before in the literature, establishing a new molecular benchmark for clinical donor selection in cell therapies.

In summary, cord blood gas and pH parameters represent important screening markers closely related to the transcriptomic programming of UC-MSCs in vitro. Although these correlations indicate a molecular change in biological quality, our study is strictly limited to transcript-level data. Ultimately, the translation of these parameters into routine clinical criteria for donor banking and therapeutic selection remains speculative until persistent functional impairment or modifications of secretome proteins are confirmed using specialized quantitative and paracrine assays.

## 6. Conclusions

Based on the conducted studies and analysis of the obtained results, we hypothesize that environmental pH may act as a molecular switch for UC-MSC function. Lower pH values likely induce a transition of cells from an actively proliferative phenotype to a structural–migratory phenotype characterized by intense reorganization of the extracellular matrix. However, this hypothesis should be confirmed in further studies, including functional assessment of cells depending on the environment and evaluation of key proteins. We also speculate that the decreased proliferative potential of UC-MSCs in acidotic conditions results from direct inhibition of the expression of key mitogens and their receptors, particularly the *EGF*, *FGFR1*, *NGF*, and *IGF1* axes. However, this also requires further investigation. Our studies demonstrate that threatened neonatal asphyxia and hypercapnia likely represent significant factors limiting the paracrine and neuroprotective potential of umbilical cord cells, as manifested by a significant decrease in growth factor expression at the transcript level. Further studies assessing key protein levels are necessary to confirm this. It also appears that, in addition to blood gas parameters, auxological factors such as birth weight and neonatal maturity (HBD) have potential significance in the angiogenic and neurotrophic profile of UC-MSCs, indicating the high plasticity of these cells in response to the fetal microenvironment. These results suggest that the acid–base status and clinical profile of the newborn should be considered as critical quality parameters when selecting and standardizing material for regenerative medicine.

## Figures and Tables

**Table 1:** Clinical characteristics of the study group and umbilical cord blood gasometry and hematological parameters.

Parameter	Microarray Group N = 6 (Expression Analysis Using Expression Microarrays)					Validation Group N = 50 (Expression Analysis by qPCR)				
	Mean	Median	Min	Max	SD	Mean	Median	Min	Max	SD
Mother’s age [years]	38.000	37.500	33.000	46.000	4.336	33.265	33.000	21.000	46.000	5.318
Pregnancy	2.333	2.000	1.000	5.000	1.506	2.184	2.000	1.000	6.000	1.302
Birth	2.000	2.000	1.000	5.000	1.549	1.898	2.000	1.000	6.000	1.159
GA [weeks]	38.500	39.000	37.000	39.000	0.837	38.184	38.000	34.000	41.000	1.550
Newborn’s body weight [g]	3096.667	3115.000	2780.000	3360.000	223.129	3251.020	3270.000	2460.000	4060.000	406.967
pH	7.367	7.360	7.310	7.440	0.045	7.331	7.340	7.030	7.450	0.070
pCO_2_ [mmHg]	42.083	41.650	34.100	50.700	5.356	44.860	42.000	30.400	77.600	8.804
PO_2_ [mmHg]	34.367	36.600	21.800	41.200	7.215	28.457	27.400	16.000	52.900	7.715
cHCO_3_ [mmol/L]	23.483	23.450	22.200	24.900	0.937	22.778	23.000	17.000	26.200	1.882
WBC [10^9^/L]	11.005	10.480	5.970	18.730	4.354	10.784	9.890	5.140	19.410	3.027
RBC [10^12^/L]	3.722	3.950	2.790	4.120	0.515	4.039	4.030	2.790	4.830	0.398
HGB [g/dL]	11.500	11.750	9.600	13.300	1.502	12.153	12.300	9.400	14.100	1.086
HCT [%]	33.017	33.300	27.500	36.800	3.632	35.394	35.700	27.500	41.300	2.948
MCV [fl]	89.233	90.350	79.800	98.600	6.375	87.878	88.000	77.400	98.600	4.654
MCH [pg]	31.083	31.700	25.800	34.400	2.963	30.190	30.600	25.500	34.400	2.057
MCHC [g/dL]	34.767	35.000	32.400	36.600	1.401	34.335	34.300	31.700	37.000	1.074
PLT [10^9^/L]	194.000	189.500	126.000	261.000	44.479	202.490	199.000	121.000	324.000	44.185

Abbreviations: GA, gestational age; WBC, white blood cells; RBC, red blood cells; HGB, hemoglobin; HCT, hematocrit; MCV, mean corpuscular volume; MCH, Mean Corpuscular Hemoglobin; MCHC, Mean Corpuscular Hemoglobin Concentration; PLT, Platelet Count.

**Table 2:** Labor course parameters and neonatal characteristics.

Group	N	%
Method of delivery		
CC	44	88
NC	6	12
Prematurity		
no	37	74
yes	13	26
Sex of the newborn		
M	26	52
F	24	48
Threatened neonatal asphyxia		
no	45	90
yes	5	10

Abbreviations: CC, cesarean section; NC, natural childbirth; M, male; F, female.

**Table 3:** Summary of the pH-dependent transcriptomic shifts and biological processes in UC-MSCs.

Biological Process	Acidic (pH < 7.35)	Physiological vs. Alkaline	Acidic vs. Alkaline	Global Trend
ECM Organization	↑	↓	↑/Remodeling	↑
Cell Adhesion	↑	↓	↑	↑
Cell Migration	↑	~	↑	↑
Cell Proliferation	↓	↑	↓	↓
Signaling Pathways	↑	Immunomodulation	↑	↑

Legend: ↑—upregulation; ↓—downregulation; ~—no significant change; ECM—Extracellular Matrix.

**Table 4:** Comparison of the expression levels of selected genes (log_10_RQ).

Gene	Mean	SD	Mean	SD	*p*-Value
**Premature (no ≥38 weeks vs. yes <38 weeks)**					
	**NO (N = 37)**		**YES (N = 13)**		
logRQ *PGF*	−0.389	0.400	−0.158	0.378	0.077
**Newborn’s body weight (>3500 g vs. <3500 g)**					
	**>3500 g (N = 15)**		**<3500 g (N = 35)**		
logRQ *FGF2*	0.206	0.244	0.083	0.207	0.046 *
logRQ *PGF*	−0.138	0.430	−0.404	0.372	0.036 *
**Threatened asphyxia (NO vs. YES)**					
	**NO (N = 45)**		**YES (N = 5)**		
logRQ *EGF*	−0.093	0.419	−0.688	0.401	0.009 *
logRQ *FGFR1*	−0.089	0.272	−0.379	0.292	0.047 *
logRQ *GDNF*	−0.614	0.453	−1.041	0.228	0.071
logRQ *IGF1*	−0.612	0.678	−1.257	0.811	0.079
logRQ *NGF*	−0.363	0.511	−0.206	0.753	0.042 *
**umbilical cord blood pH (>7.35 vs. <7.35)**					
	**<7.35 (N = 25)**		**>7.35 (N = 25)**		
logRQ *EGF*	−0.288	0.459	0.006	0.387	0.022 *
**umbilical cord blood pH (>7.3 vs. ≤7.3)**					
	**>7.3 (N = 39)**		**<7.3 (N = 11)**		
logRQ *GDNF*	−0.699	0.420	−0.436	0.556	0.018 *
logRQ *IGF1*	−0.562	0.599	−1.118	0.957	0.031 *
logRQ *NGF*	−0.243	0.402	−0.827	0.749	0.002 *
**umbilical cord blood pCO_2_ [mmHg] (35–45 vs. >45)**					
	**35–45 (N = 28)**		**>45 (N = 17)**		
logRQ *EGF*	−0.013	0.374	−0.434	0.387	0.001 *
logRQ *FGFR1*	−0.061	0.205	−0.223	0.345	0.050 *
logRQ *HGF*	0.083	0.507	−0.358	0.523	0.006 *
**WBC [10^9^/L] (<11 vs. 11–15)**					
	**<11 (N = 24)**		**11–15 (N = 19)**		
logRQ *EGF*	0.013	0.499	−0.334	0.317	0.012 *
logRQ *HGF*	0.050	0.494	−0.254	0.618	0.076
logRQ *NES*	0.308	0.339	0.027	0.398	0.014 *
logRQ *NGF*	−0.198	0.366	−0.453	0.556	0.072
logRQ *PGF*	−0.236	0.424	−0.450	0.351	0.078
**HGB [g/dL] (>11.5 vs. <11.5)**					
	**>11.5 (N = 35)**		**<11.5 (N = 15)**		
logRQ *NES*	0.128	0.407	0.373	0.229	0.040 *

Values in boldface and red text, marked with an asterisk (*), indicate statistically significant values *p* < 0.05 *t*-student Test.

**Table 5:** Correlations between log_10_RQ values of individual genes encoding growth factors.

Parameter	*logRQ EGF*	*logRQ FGF2*	*logRQ FGFR1*	*logRQ FGFR3*	*logRQ GDNF*	*logRQ HGF*	*logRQ IGF1*	*logRQ NES*	*logRQ NGF*	*logRQ PGF*
**Inter-Genic Correlations**										
*logRQ EGF*	1.000	0.080	0.437 *	0.246	0.446 *	0.543 *	0.165	0.258	0.004	0.268
*logRQ FGF2*	0.080	1.000	0.237	0.177	0.245	−0.174	0.096	0.129	0.300 *	0.451 *
*logRQ FGFR1*	0.437 *	0.237	1.000	0.631 *	0.458 *	0.282 *	0.509 *	0.142	0.403 *	0.527 *
*logRQ FGFR3*	0.246	0.177	0.631 *	1.000	0.468 *	0.015	0.412 *	0.109	0.501 *	0.590 *
*logRQ GDNF*	0.446 *	0.245	0.458 *	0.468 *	1.000	0.171	0.312 *	0.042	0.069	0.543 *
*logRQ HGF*	0.543 *	−0.174	0.282 *	0.015	0.171	1.000	0.170	−0.018	0.053	−0.065
*logRQ IGF1*	0.165	0.096	0.509 *	0.412 *	0.312 *	0.170	1.000	−0.138	0.357 *	0.362 *
*logRQ NES*	0.258	0.129	0.142	0.109	0.042	−0.018	−0.138	1.000	−0.049	0.294 *
*logRQ NGF*	0.004	0.300 *	0.403 *	0.501 *	0.069	0.053	0.357 *	−0.049	1.000	0.404 *
*logRQ PGF*	0.268	0.451 *	0.527 *	0.590 *	0.543 *	−0.065	0.362 *	0.294 *	0.404 *	1.000
**Perinatal and Maternal Clinical Characteristics**										
Mother’s age [years]	0.069	−0.037	−0.189	−0.006	0.170	−0.075	−0.031	0.018	−0.198	−0.118
Pregnancy	−0.038	−0.091	−0.343 *	−0.035	0.085	−0.237	−0.096	0.264	−0.132	0.000
Birth	−0.100	−0.089	−0.344 *	−0.031	0.062	−0.288 *	−0.006	0.197	−0.064	−0.043
GA [weeks]	−0.023	−0.036	0.023	0.054	−0.002	−0.097	−0.101	−0.344 *	−0.210	−0.223
Newborn’s body weight [g]	0.077	0.153	−0.131	−0.055	−0.089	−0.146	−0.154	0.014	−0.111	0.087
**Umbilical Cord Blood Gasometric and Hematological Indices**										
pH	0.303 *	−0.005	0.283 *	0.149	0.025	0.201	0.305 *	0.126	0.390 *	0.128
pCO_2_ [mmHg]	−0.304 *	−0.058	−0.329 *	−0.201	−0.009	−0.229	−0.215	−0.080	−0.444 *	−0.116
PO_2_ [mmHg]	0.180	−0.169	0.205	−0.018	−0.179	0.078	−0.086	0.172	−0.093	−0.105
cHCO_3_ [mmol/L]	−0.151	−0.151	−0.194	−0.147	0.000	−0.114	0.151	0.040	−0.140	−0.060
WBC [10^9^/L]	−0.157	−0.112	0.011	0.030	−0.054	−0.054	−0.135	−0.159	−0.221	−0.131
RBC [10^12^/L]	−0.040	0.135	−0.100	−0.074	0.018	0.007	−0.097	−0.229	0.136	0.058
HGB [g/dL]	0.024	0.181	0.103	0.084	0.142	0.057	0.152	−0.197	0.171	0.130
HCT [%]	0.088	0.109	0.061	−0.039	0.145	0.085	0.084	−0.216	0.068	0.078
MCV [fl]	0.211	−0.087	0.264	0.074	0.183	0.098	0.283	0.081	−0.163	−0.010
MCH [pg]	0.100	0.038	0.271	0.225	0.158	0.045	0.312 *	0.070	0.006	0.062
MCHC [g/dL]	−0.155	0.227	0.136	0.355 *	0.039	−0.079	0.197	0.016	0.299 *	0.167
PLT [10^9^/L]	−0.099	−0.190	−0.094	−0.074	−0.308 *	−0.154	0.052	−0.265	0.033	−0.218

Pearson correlations, values in boldface and red text, marked with an asterisk (*), indicate statistically significant correlations (*p* < 0.05). Abbreviations: GA, gestational age; WBC, white blood cells; RBC, red blood cells; HGB, hemoglobin; HCT, hematocrit; MCV, mean corpuscular volume; MCH, Mean Corpuscular Hemoglobin; MCHC, Mean Corpuscular Hemoglobin Concentration; PLT, Platelet Count.

**Table 6:** Validation of Microarray Findings through qPCR Analysis: Consistency of Gene Expression Patterns and Biological Pathways.

Gene/Pathway	Microarray Result (pH < 7.35)	qPCR Result (pH Correlation/Asphyxia)	Consistency
FGFR Signaling	↓ Expression of *FGFR2*, *FGF5/7/10*	↓ Expression of *FGFR1* (r = 0.28, *p* < 0.05)	High
EGF Signaling	↓ Proliferative pathways	↓ Expression of *EGF* (r = 0.30, *p* < 0.05)	High
Growth Factors	↓ Enzyme-linked receptor signaling	↓ *NGF*, *IGF1* in low pH and asphyxia	High
Proliferation	↓ Inhibition of cell division (GO:BP)	↓ Key mitogens (*EGF*, *IGF1*)	High

Legend: ↓—downregulation/decrease; r—Spearman’s correlation coefficient; *p*—statistical significance; GO:BP—Gene Ontology Biological Process.

## Data Availability

The original contributions presented in this study are included in the article/Supplementary Materials. Further inquiries can be directed to the corresponding author.

## Supplementary Materials

The following supporting information can be downloaded at: https://www.mdpi.com/article/10.3390/cells15121076/s1, Table S1: Global effect of pH—Selected biologically significant pathways. Results of functional enrichment analysis (g:Profiler) for genes differentiating pH groups. Selected biologically significant pathways with example genes belonging to individual categories (intersection). *p*_adj_ values correspond to adjusted significance levels (FDR); Table S2: Biologically Relevant Pathways, overexpressed genes for group comparison: <7.35 vs. 7.35–7.39. Functional enrichment analysis revealed significant changes in pathways related to extracellular matrix organization, cell adhesion, and cytoskeletal reorganization. Processes such as extracellular matrix organization, cell adhesion, and cell junction organization were particularly enriched (Log2FC > 1, 176 genes); Table S3: Functional enrichment analysis. Selected biologically significant (GO) pathways with representative genes for genes with decreased expression for pH < 7.35 vs. pH = 7.35–7.39. P_adj_ values represent adjusted significance ratios (FDR). (LOG2FC < −1; 241 genes); Table S4: (a) Functional enrichment analysis of upregulated (UP) genes comparing pH 7.4 vs. pH 7.35–7.39 samples. Selected biologically relevant pathways are presented along with the genes belonging to a given category (intersection). *p*_adj_ values represent the adjusted significance level (FDR). (Log2FC > 1, 6 genes). (b) Functional enrichment analysis of downregulated genes (DOWN) comparing pH 7.4 versus pH 7.35–7.39 samples. Selected biologically relevant pathways (GO and CORUM) are presented along with the genes belonging to a given category (intersection). *p*_adj_ values represent adjusted significance levels (FDR). (LOG2FC < −1, 21 genes); Table S5: Functional enrichment analysis for genes with increased expression in samples with pH < 7.35 versus pH ≥ 7.4 revealed significant changes in pathways related to receptor signaling, extracellular matrix organization, and cell adhesion. Processes such as enzyme-linked receptor protein signaling pathway, extracellular matrix, and cell adhesion were particularly enriched. (FDR < 0.25, Log2FC > 1, 173 genes); Table S6: Functional enrichment analysis for genes with decreased expression in samples with pH < 7.35 versus pH ≥ 7.4 revealed significant changes in pathways related to cell interactions with the extracellular matrix and cell surface. Collagen binding, glycosaminoglycan binding, and extracellular space were particularly enriched. (FDR < 0.25, LOG2FC < −1, 201 genes).
