# Supplementary material for: Umbilical Cord Blood Gasometry and pH as Key Regulators of Growth Factor Expression Profile in Umbilical Cord-Derived Mesenchymal Stromal Cells (UC-MSCs)
Source: Cells. 2026 Jun 13;15(12):1076. doi: 10.3390/cells15121076 (PMC13297315; doi:10.3390/cells15121076)
Supplement: Supplementary file 1 [file cells-15-01076-s001.zip › cells-4301623-supplementary.pdf]

Supplementary Table S1. Global effect of pH - Selected biologically significant pathways. Results of functional enrichment analysis (g:Profiler) for genes differentiating pH groups. Selected biologically significant pathways with example genes belonging to individual categories (intersection). p_adj values correspond to adjusted significance levels (FDR).

Source	Term Name	padj	GO ID	Genes
GO:BP	enzyme-linked receptor protein signaling pathway	3.44E-12	GO:0007167	*COMP, DCN, EFNB2, EPHA3, FGF5, FGF7, FGF10,* ***FGFR2***
GO:MF	signaling receptor binding	6.72E-04	GO:0005102	*COMP, CSF1, EFNB2, FGF5, FGF7, FGF10, HBEGF, VEGFA*
GO:BP	positive regulation of protein phosphorylation	1.17E-02	GO:0001934	*CSF1, FGF5, FGF7, FGF10, HBEGF, IL1B, VEGFA*
GO:BP	peptidyl-tyrosine phosphorylation	1.67E-02	GO:0018108	*DDR1, EPHA3, FGF7, FGF10,* ***FGFR2****, FLT1, VEGFA*
GO:BP	positive regulation of canonical Wnt signaling pathway	4.19E-02	GO:0090263	*BAMBI, DKK2, FGF10,* ***FGFR2****, GPC3, SFRP1*
GO:BP	extracellular matrix organization	1.91E-05	GO:0030198	*COL15A1, COMP, DPT, FMOD, LUM, MMP1*
GO:MF	extracellular matrix structural constituent	1.37E-06	GO:0005201	*COL15A1, COMP, DCN, FMOD, LUM, PRELP*
GO:BP	cell adhesion	7.35E-07	GO:0007155	*AOC3, CDH7, COMP, DDR1, EFNB2, EPHA3*
GO:BP	regulation of cell-substrate adhesion	2.76E-02	GO:0010810	*DDR1, EPHA3, HAS2, MMP12, PDPN*
GO:MF	collagen binding	3.33E-02	GO:0005518	*COMP, DDR1, LUM, MMP12*
GO:BP	circulatory system process	1.11E-02	GO:0003013	*COMP, FOXC2, HBEGF, STC1, VEGFA*
GO:BP	response to hypoxia	1.37E-02	GO:0001666	***FGFR2****, MYC, STC1, TGFB3, VEGFA*
GO:BP	secretion	2.56E-02	GO:0046903	*FGF7, FGF10, IL1B, PRLR, TGFB3, VEGFA*

Supplementary Table S2. Biologically Relevant Pathways, overexpressed genes for group comparison: <7.35 vs 7.35-7.39. Functional enrichment analysis revealed significant changes in pathways related to extracellular matrix organization, cell adhesion, and cytoskeletal reorganization. Processes such as extracellular matrix organization, cell adhesion, and cell junction organization were particularly enriched (Log2FC>1, 176 genes).

**Source**	**Term Name**	**padj**	**GO ID**	**Genes**
GO:MF	extracellular matrix structural constituent	6.40E-07	GO:0005201	*FMOD, DCN, PRELP, DPT, COL15A1, SPON1, LUM, COMP*
GO:BP	cell adhesion	1.24E-09	GO:0007155	*PRLR, CADM3, SORBS1, ATP1B1, AOC3, CDH7, PTPRT*
GO:BP	extracellular matrix organization	1.36E-02	GO:0030198	*FMOD, MMP1, DPT, CRISPLD2, COL15A1, LUM, COMP*
GO:CC	extracellular matrix	2.77E-11	GO:0031012	*FMOD, MMP1, GPC3, DCN, PRELP, FGF10, DPT, COL15A1*
GO:BP	cell junction organization	5.58E-04	GO:0044330	*SORBS1, FILIP1, CDH7, PTPRT, PCDHB14*
GO:CC	cell junction	1.21E-06	GO:0030054	*DGKI, CADM3, SORBS1, FILIP1, FZD3, ATP1B1*
GO:BP	actin filament-based process	3.35E-02	GO:0030029	*SORBS1, IQGAP2, FHOD3, MYH11, EPB41L3*
GO:BP	actomyosin structure organization	1.49E-02	GO:0031032	*SORBS1, IQGAP2, FHOD3, MYH11, CNN1*
GO:BP	response to endogenous stimulus	1.36E-08	GO:0009719	*WFDC1, DGKI, GPC3, FGF10, IQGAP2*
GO:BP	positive regulation of canonical Wnt signaling pathway	9.33E-03	GO:0090263	*GPC3, FGF10, FGFR2, BAMBI, DKK2, SFRP1*
GO:MF	transforming growth factor beta receptor binding	1.33E-02	GO:0005160	*RASL11B, BAMBI, TGFB3, SMAD6*
GO:BP	blood circulation	4.63E-02	GO:0008015	*ATP1B1, COMP, STC1, KCNMB1, PLN*

Additionally, changes were identified in pathways related to cell structure, including actin filament-based process and actomyosin structure organization, as well as in signaling processes such as Wnt signaling pathway and TGF-beta receptor binding.

Supplementary Table S3. Functional enrichment analysis. Selected biologically significant (GO) pathways with representative genes for genes with decreased expression for pH <7.35 vs. pH = 7.35–7.39. Padj values represent adjusted significance ratios (FDR). (LOG2FC < -1; 241 genes)

**Source**	**Term Name**	**padj**	**GO ID**	**Genes**
GO:BP	nuclear division	3.33E-08	GO:0000280	*CDK1, CDC20, BUB1, AURKA, CENPF*
GO:BP	cell division	8.93E-04	GO:0051301	*CDK1, CDC20, AURKA, TOP2A*
GO:BP	chromosome localization	3.87E-03	GO:0050000	*CENPF, TOP2A, BUB1*
GO:CC	spindle	8.10E-05	GO:0005819	*AURKA, TPX2, KIF11*
GO:MF	tubulin binding	5.02E-05	GO:0015631	*KIF11, TPX2, MAPRE1*
GO:BP	cell population proliferation	1.28E-02	GO:0008283	*CDK1, MYBL2, TOP2A*
GO:BP	DNA binding, bending	1.64E-02	GO:0008301	*HMGB2, TOP2A*
GO:BP	intracellular signal transduction	7.80E-03	GO:0035556	*MAPK, AKT, SRC*
GO:BP	cell adhesion	9.93E-03	GO:0007155	*ITGB1, CDH2, FN1*
GO:BP	cell junction organization	1.07E-02	GO:0034330	*CDH2, CTNNB1*
GO:BP	cell migration	2.82E-02	GO:0016477	*ITGB1, FN1*
GO:BP	response to oxygen levels	2.67E-02	GO:0070482	*HIF1A, VEGFA*

Supplementary Table S4a. Functional enrichment analysis of upregulated (UP) genes comparing pH 7.4 vs. pH 7.35–7.39 samples. Selected biologically relevant pathways are presented along with the genes belonging to a given category (intersection). p_adj values represent the adjusted significance level (FDR). (Log2FC>1, 6 genes)

**Source**	**Term Name**	**padj**	**GO ID**	**Genes**
Reactome	TLR3 deficiency - HSE	4.99E-02	R-HSA-566	*TLR3*

Supplementary Table S4b. Functional enrichment analysis of downregulated genes (DOWN) comparing pH 7.4 versus pH 7.35–7.39 samples. Selected biologically relevant pathways (GO and CORUM) are presented along with the genes belonging to a given category (intersection). p_adj values represent adjusted significance levels (FDR). (LOG2FC < -1, 21 genes)

**Source**	**Term Name**	**padj**	**GO ID**	**Genes**
GO:CC	extracellular matrix	8.09E-04	GO:0031012	*MMP1, FMOD, DPT, HTRA3, ANGPTL4*
GO:CC	collagen-containing extracellular matrix	2.67E-03	GO:0062023	*FMOD, DPT*
CORUM	ANGPTL4-LRP6-SDC2 complex	4.99E-02	CORUM:7289	*ANGPTL4*

Supplementary Table S5. Functional enrichment analysis for genes with increased expression in samples with pH <7.35 versus pH ≥7.4 revealed significant changes in pathways related to receptor signaling, extracellular matrix organization, and cell adhesion. Processes such as enzyme-linked receptor protein signaling pathway, extracellular matrix, and cell adhesion were particularly enriched. (FDR<0.25, Log2FC>1, 173 genes)

**Source**	**Term Name**	**padj**	**GO ID**	**Genes**
GO:BP	enzyme-linked receptor protein signaling pathway	8.76E-11	GO:0007167	*FGF10, FGFR2, TGFB3, BAMBI, GPC3*
GO:CC	extracellular matrix	2.24E-08	GO:0031012	*FMOD, DCN, LUM, COL15A1, DPT*
GO:MF	extracellular matrix structural constituent	1.46E-05	GO:0005201	*FMOD, DCN, LUM, PRELP*
GO:BP	cell adhesion	1.82E-05	GO:0007155	*CDH7, AOC3, SORBS1*
GO:CC	cell junction	2.06E-06	GO:0030054	*SORBS1, FILIP1*
GO:BP	cell junction organization	2.18E-03	GO:0034330	*CDH7, SORBS1*
GO:BP	cell migration	4.86E-03	GO:0016477	*SORBS1, IQGAP2*
GO:MF	transforming growth factor beta receptor binding	6.39E-03	GO:0005160	*TGFB3, BAMBI*
GO:BP	positive regulation of cell population proliferation	1.32E-02	GO:0008284	*FGF10, TGFB3*
GO:BP	growth	2.11E-03	GO:0040007	*FGF10, GPC3*

Additionally, changes in processes related to cell migration and the organization of intercellular connections, including cell migration and cell junction organization, as well as in signaling pathways such as TGF-beta receptor binding, were identified.

Supplementary Table S6. Functional enrichment analysis for genes with decreased expression in samples with pH <7.35 versus pH ≥7.4 revealed significant changes in pathways related to cell interactions with the extracellular matrix and cell surface. Collagen binding, glycosaminoglycan binding, and extracellular space were particularly enriched. (FDR<0.25, LOG2FC < -1, 201 genes)

**Source**	**Term Name**	**padj**	**GO ID**	**Genes**
GO:MF	collagen binding	1.65E-03	GO:0005518	*COMP, LUM, MMP1*
GO:MF	glycosaminoglycan binding	7.69E-03	GO:0005539	*LUM, PRELP*
GO:CC	extracellular space	6.80E-04	GO:0005615	*MMP1, ANGPTL4, DPT*
GO:CC	cell surface	2.62E-03	GO:0009986	*ANGPTL4, AOC3*
GO:BP	cell surface receptor signaling pathway	5.98E-03	GO:0007166	*FGFR2, TGFB3*
GO:BP	positive regulation of cell population proliferation	4.96E-03	GO:0008284	*FGF10, TGFB3*
GO:BP	positive regulation of cell migration	4.18E-02	GO:0030335	*SORBS1*

Additionally, changes in receptor signaling pathways and cell functional processes were identified, including cell surface receptor signaling pathway, positive regulation of cell population proliferation and positive regulation of cell migration.
